# New isoxazole-based heterocyclic hybrids with dual antimicrobial and antioxidant bioactivity: integrated synthesis, *in vitro* assessment, and computational exploration

**DOI:** 10.1039/d6ra01005a

**Published:** 2026-03-10

**Authors:** Aziz Arzine, Soumia Ait Assou, Lamiae El Bouamri, Mohammed Chalkha, Asmae Nakkabi, Samir Chtita, Reem M. Aljowaie, Mourad A. M. Aboul-Soud, Mohammed El Hassouni, John P. Giesy, Mohamed El Yazidi

**Affiliations:** a Engineering Laboratory of Organometallic, Molecular Materials and Environment, Faculty of Sciences Dhar EL Mahraz, Sidi Mohamed Ben Abdellah University P.O. Box 1796 (Atlas) 30000 Fez Morocco arzineaziz@gmail.com mohammed.chalkha1@usmba.ac.ma; b Biotechnology, Environment, Agri-Food and Health Laboratory, Faculty of Sciences Dhar El Mahraz, Sidi Mohamed Ben Abdellah University BP. 1796, Atlas Fez Morocco; c Laboratory of Analytical and Molecular Chemistry, Faculty of Sciences Ben M'Sik, Hassan II University of Casablanca Casablanca B.P 7955 Morocco; d Laboratory of Materials Engineering for the Environment and Natural Resources, Faculty of Sciences and Techniques, University of Moulay Ismail of Meknes B.P 509, Boutalamine 52000 Errachidia Morocco; e Department of Botany and Microbiology, College of Science, King Saud University P.O. Box 2455 Riyadh 11451 Saudi Arabia; f Center of Excellence in Biotechnology Research, College of Applied Medical Sciences, King Saud University Riyadh 11433 Saudi Arabia maboulsoud@ksu.edu.sa; g Department of Veterinary Biomedical Sciences, Toxicology Centre, Western College of Veterinary Medicine, University of Saskatchewan Saskatoon SK S7N 5B4 Canada; h Department of Integrative Biology, Center for Integrative Toxicology, Michigan State University East Lansing MI 48824 USA; i Department of Environmental Sciences, Baylor University Waco 76706 USA

## Abstract

In the present study, novel isoxazole-based hybrid compounds 5a–h were synthesized with satisfactory yields. Their structure was confirmed by FT-IR, NMR (^1^H, ^13^C, 2D), and HRMS. The antimicrobial properties of this class of compounds were thoroughly investigated *in vitro* against a variety of Gram-positive and Gram-negative bacteria as well as different fungi, both yeast and molds. Their antioxidant ability was also assessed by molybdate reduction assay. The compounds showed excellent antifungal activity, particularly against *Aspergillus niger* and *Fusarium oxysporum*, equivalent to that of fluconazole. Among the series, 5d was the most potent antibacterial agent against *Escherichia coli* (minimal inhibitory concentration, MIC = 2.582 µmol mL^−1^), whereas the highest potency against *Bacillus subtilis* was found for 5h (MIC = 0.083 µmol mL^−1^) which was comparable to the efficacy of ampicillin. With respect to the antifungal activity, 5h showed the lowest MIC value against *Candida albicans* (MIC = 0.083 µmol mL^−1^) and *A. niger* (MIC = 0.083 µmol mL^−1^), and 5g was the most effective against *Aspergillus flavus* (MIC = 0.044 µmol mL^−1^). All hybrids were more effective as compared to fluconazole against *F. oxysporum* (MIC range: 0.43–0.94 µmol mL^−1^). Furthermore, assessment of their antioxidant potential shows that compounds 5e and 5g exhibit excellent reducing potency. Molecular docking and dynamic simulations demonstrated that compounds 5a–h, especially 5d, interact strongly and stably with key bacterial and fungal proteins, forming hydrogen bonds, hydrophobic interactions, and water bridges, with minimal root-mean-square fluctuations (RMSF), confirming the structural integrity of the complexes. Additionally, *in silico* predictions of Absorption, Distribution, Metabolism, Excretion, and Toxicity (ADME-Tox) analysis predicted 5d to possess an optimal profile, with high gastrointestinal absorption and minimal toxicity, highlighting it as the lead candidate for the development of broad-spectrum antimicrobial drugs.

## Abstract 2

This article reports comprehensive research on the synthesis of novel isoxazole-based heterocyclic hybrids, their characterization and the evaluation of their antimicrobial and antioxidant properties, employing both *in vitro* and *in silico* approaches.

## Graphical abstract

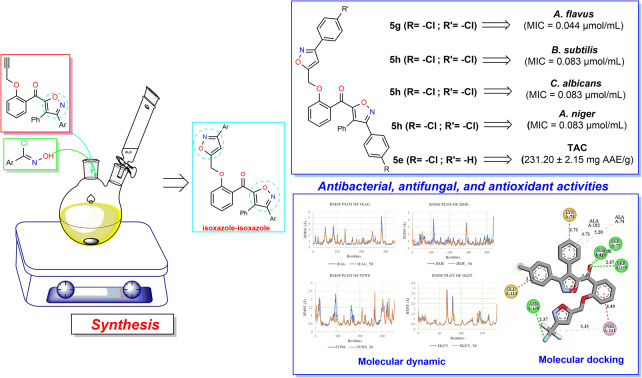

## 1. Introduction

Nitrogen-containing heterocycles represent a significant class of bioactive compounds that play a fundamental role in the medicinal chemistry field.^1–3^ Among these, azoles constitute a subclass of five-membered aromatic heterocycles, comprising at least one nitrogen atom and often one or more additional heteroatoms, such as oxygen or sulfur.^4–7^

The isoxazole pharmacophore, a five membered heterocyclic ring containing two adjacent heteroatoms (oxygen and nitrogen), plays a central role in many pharmaceutical agents.^8–10^ The importance of this structural element is highlighted in its role as a key mediator in the synthesis of several biologically active molecules.^11–18^ At the molecular level, the isoxazole residue can establish various non-covalent interactions, including nitrogen and oxygen hydrogen bonds, pi–pi, and hydrophobic interactions (cLogP = 0.334 at pH = 7.4).^19^ These properties improve isoxazole's extensive pharmacological applications, as shown by its incorporation into various therapeutic drugs, including β-lactam antibiotics (cloxacillin and dicloxacillin) and antibacterial sulfonamides (sulfisoxazole and sulfamethoxazole), as well as anti-inflammatory agents such as valdecoxib (a selective COX-II inhibitor) and leflunomide (a disease-modifying immunosuppressant (DMARD)).^6,20–24^ The isoxazole heterocycle has been found in numerous biologically active natural and synthetic substances, including the drugs cycloserine, acivicine, and muscimol. These compounds were originally obtained from microorganisms, higher plants and marine sponges.^25–27^ In addition, a wide range of therapeutic potential of isoxazole derivatives has also been reported, including antifungal,^28,29^ antiviral,^30,31^ antihistamine,^32^ antimicrobial,^11,13–15^ antioxidant,^16,33^ anticancer,^17,34^ and anti-inflammatory^35,36^ effects. Apart from their importance in medicine, these compounds are widely used in agriculture and industry,^8^ as insecticides,^37^ herbicides,^38^ fungicides,^39^ and as anti-corrosion coating agents.^40^ This broad-spectrum of potential applications highlights the isoxazole nucleus as a highly promising component at the interface between organic chemistry, biology and materials science.

Molecule hybridization is, currently, considered as one of the most innovative and rational approaches for designing new chemical entities exhibiting multiple therapeutic applications.^41–43^ This hybridization strategy involves designing a substance that contains two or more pharmacophores from different compounds, with each pharmacophore contributing to specific biological effects or physicochemical proprieties.^44–46^ This kind of combination has been found to enhance the biological activity of these molecules, as well as their selectivity and pharmacokinetic profile. Such combination has also been demonstrated to attenuate side effects and drug resistance.^44,47,48^ Given these considerations, the isoxazole moiety was considered as a crucial bioactive scaffold in developing new hybrid molecules. To increase their activity and to the investigate the formation of new synergistic effects.^12,14^ In the last decades, many hybrids have been synthesized and investigated, in which a variety of bioactive moieties were linked including isoxazole–(iso)oxazole types standing out as a significant example of heterocyclic hybrids. The molecules obtained through these hybridizations have demonstrated various pharmacological activities, including anticancer, antibacterial, anti-inflammatory, immunosuppressive, antitubercular, as well as antiviral and antifungal activities.^14,41,49–51^

In the present study, an efficient synthesis is described for developing new hybrids that contain di- and trisubstituted isoxazole moieties. This approach is based on the idea that the pharmacophoric elements act synergistically leading to molecules that display improved biologically effect. The newly synthesized hybrids were subjected to a combined assessment of their antioxidant and antimicrobial activity, *in vitro*, and the results indicated encouraging findings. Concurrently, a series of *in silico* studies, including molecular docking, ADME-Tox and drug-likeness prediction, and molecular dynamics simulation (MDS) were conducted to support the results obtained from the, *in vitro*, experiments tests. Structure-based approaches have been used to study binding interactions, evaluate pharmacokinetic features, and verify the stability of the subsequent ligand–protein complexes. Consequently, the experimental and theoretical investigations provide deeper understanding into the modification of new isoxazole–isoxazole hybrids.

## 2. Results and discussion

### 2.1. Chemistry

**Scheme 1:**
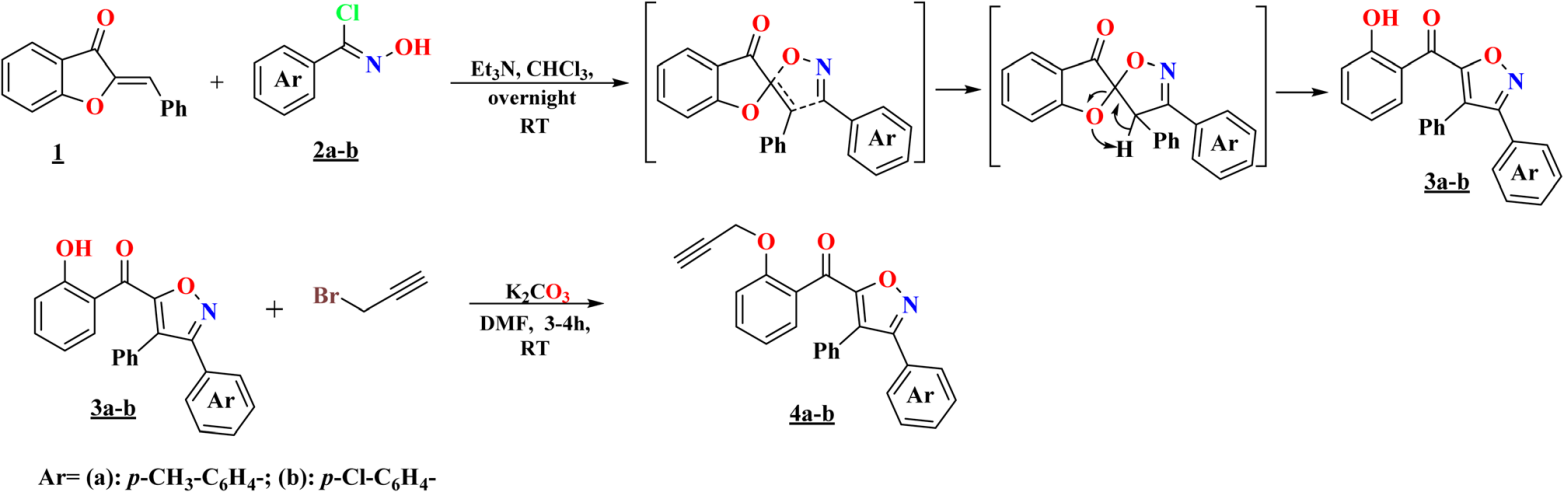
Synthesis pathway of *o*-propargyl isoxazoles 4a–b.

The *o*-propargylated isoxazoles 4a–b have been prepared from aroylisoxazoles 3a–b, which were obtained by a 1,3-dipolar cycloaddition reaction involving 2-benzylidene-3(2*H*)-benzofuranone 1 and 1,3-arylnitriloxide dipoles 2a–b. These 1,3-dipoles are generated *in situ* by the action of triethylamine on the corresponding chlorinated aldoximes. The reaction, due to a prototropic-1,3 rearrangement, does not lead to the expected spiroisoxazolines, the result is instead aroylisoxazoles. The high reactivity of the hydroxyl group of the substrate was then exploited in an *o*-alkylation reaction with propargyl bromide, in the presence of K_2_CO_3_ as a base and DMF as a solvent at room temperature, leading to the formation of the corresponding *o*-propargylated isoxazoles 4a–b (Scheme 1).^11,16^

**Scheme 2:**
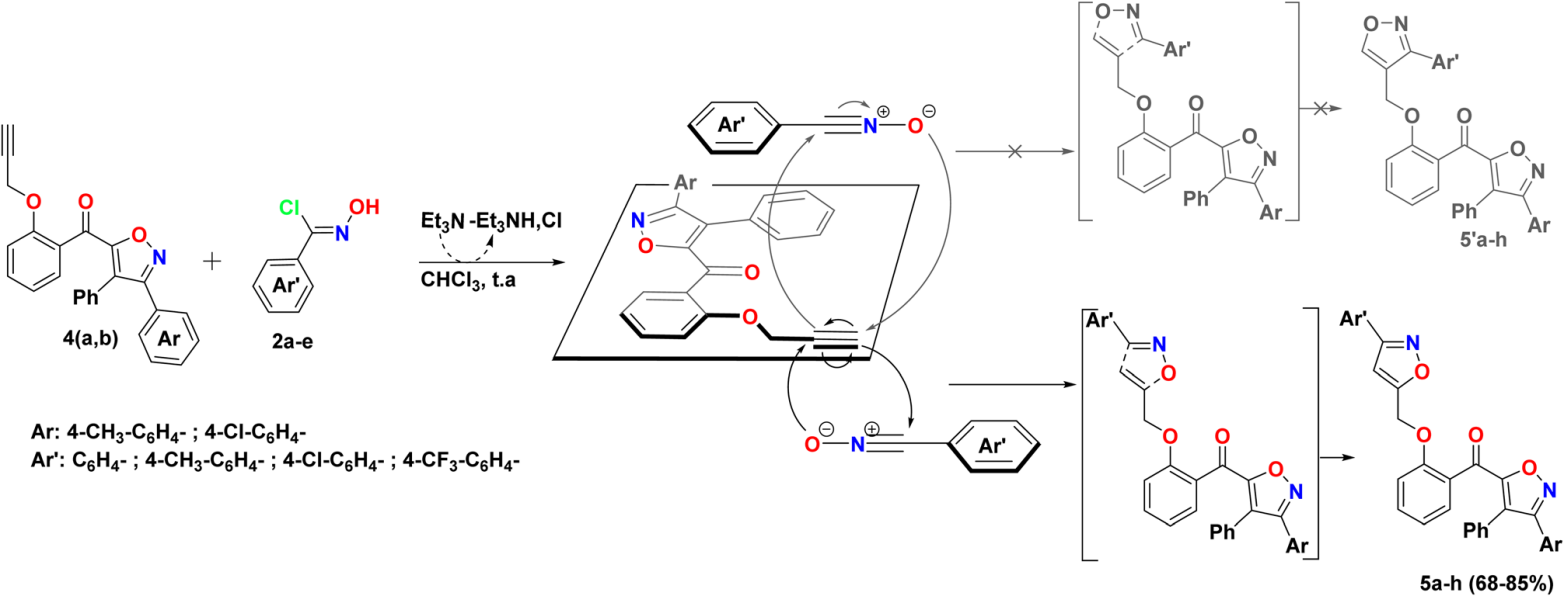
Synthesis of a series of novel isoxazole–isoxazole hybrids 5a–h.

The previously synthesized *o*-propargylated isoxazoles 4a–b, were used as dipolarophiles in 1,3-dipolar cycloaddition reactions with arylnitriloxide dipoles 2a–d, which were generated *in situ* from the corresponding chlorinated aldoximes under the action of triethylamine in chloroform at room temperature. The course of these reactions was marked by a high degree of regioselectivity, culminating in the exclusive formation of 3,5-disubstituted isoxazole rings. Consequently, a novel series of hybrid systems 5a–h was obtained, comprising two 3,5-di- and 3,4,5-trisubstituted isoxazole rings, linked by a methoxybenzoyl motif serving as a structural linker, with satisfactory yields (Scheme 2). This remarkable regioselectivity underscores the controlled reactivity of arylnitriloxide dipoles toward *o*-propargylated dipolarophiles, thereby providing an efficient synthetic route for the construction of complex heterocyclic structures with high biological potential.

A series of in-depth spectroscopic analyses were conducted to confirm the structures of the newly synthesized hybrid compounds 5a–h, including FT-IR, NMR (^1^H, ^13^C, and 2D), and HRMS. The combined evaluation of the results confirmed the structures of the obtained compounds unambiguously.

The hybrid compound 5b has been selected as a representative example from the series 5a–h. An examination of its IR spectrum (Fig. S9) revealed a band at 3115 cm^−1^ associated with the absorption of the C4–H bond of the isoxazole ring. A second absorption band of the aromatic C–H stretching vibration occurred at approximately 3112 cm^−1^. Two bands at around 2925 and 2871 cm^−1^ are weak (low-intensity) and are assigned to aliphatic C–H stretching. A strong band at approximately 1661 cm^−1^ corresponds to the stretching vibration of the C=O carbonyl function. The ^1^H NMR spectrum of the compound (Fig. S6) displays two singlet peaks at 2.31 and 2.41 ppm, indicating the presence of protons from two methyl groups (–CH_3_). A singlet resonance at 5.07 ppm was assigned to the methylene protons (–O–CH_2_–). It is evident that an additional singlet signal at 6.39 ppm is consistent with the isoxazole proton (–C_4_H–). The ^13^C NMR spectrum (Fig. S7) of the sample exhibited two signals at 21.33 and 21.44 ppm, attributed to methyl carbons (–CH_3_). A signal at 61.90 ppm corresponds to the methylene carbon atom (–O–CH_2_–). A signal at 101.22 ppm is attributed to the disubstituted isoxazole carbon (C4). Two additional signals emerge at 167.07 and 156.59 ppm, corresponding respectively to the carbons (C5) of the di- and trisubstituted isoxazole nuclei. The signal at 183.50 ppm is attributed to the carbonyl function carbon (C=O). The 2D-HMBC (^1^H–^13^C) NMR spectrum (Fig. S8) of compound 5b revealed long-distance correlations (2*J* and 3*J*). The obtained correlations confirm the regioselectivity of the cycloaddition, thereby establishing unequivocally that the isolated compound corresponds to the 3,5-disubstituted regioisomer and not to the 3,4-disubstituted isomer. The methyl protons Ha (5.07 ppm) exhibited two cross-correlations with two characteristic carbons of the isoxazole ring: C4 (101.22 ppm) and C5 (167.07 ppm). The aforementioned protons also exhibit a correlation with carbon C1′ (156.59 ppm), thereby suggesting a direct spatial proximity. In addition, the Hb proton of the isoxazole (6.04 ppm) showed two significant correlations with C3 (162.44 ppm) and C5 (167.09 ppm). The correlations confirmed their significance in the isoxazole ring, particularly between C5 and the Ha and Hb protons. The correlations obtained were further confirmed by the literature, which accurately confirmed that the cycloadduct obtained is a 3,5-disubstituted regioisomer.^12,52–55^ The structures of the synthesized hybrid compounds were confirmed by HRMS. For compounds 5b, taken as a representative example, its spectrum (Fig. S10) shows a molecular ion peak at *m*/*z* = 549.1456, which corresponds exactly to the sodium ion mass [M + Na]^+^ at *m*/*z* = 549.1903. A second peak, at *m*/*z* = 527.19299, is attributed to the protonated molecular ion, [M + H]^+^, and matches the calculated value well (*m*/*z* = 527.19708). Additionally, it demonstrates typical fragments of the molecular ion; with several examples illustrated in Scheme S1.

### 2.2. Antimicrobial activity

The newly synthesized 5a–h hybrids were subjected to, *in vitro*, antimicrobial assay. This study aimed to evaluate the potential antibacterial activity of these hybrids against three strains: two Gram-positive bacteria; *Staphylococcus aureus* ATCC 29213 and *Bacillus subtilis* ATCC 6633 and one Gram-negative bacterium *Escherichia coli* K12. Concurrently, they were evaluated as antifungal agents against four fungal strains: the yeast *Candida albicans* ATCC 10231 and the molds *Aspergillus niger* MTCC 282, *Aspergillus flavus* MTCC 9606, and *Fusarium oxysporum* MTCC 9913. The experimental tests were carried out in accordance with the MIC assay method, with previous protocols being adapted through certain modifications to optimize the testing conditions.^3,56–58^ Ampicillin was used as a positive control for both Gram-positive and Gram-negative bacteria, while fluconazole and amphotericin B served as positive controls for antifungal activity. DMSO was employed as a negative control to verify the absence of solvent effect.

As shown in Table 1, the MICs (in µmol mL^−1^) vary depending on the nature of the aromatic substituents, Ar (4-CH_3_–C_6_H_4_–; 4-Cl–C_6_H_4_–) and Ar′ (C_6_H_5_–; 4-CH_3_–C_6_H_4_–; 4-Cl–C_6_H_4_–; 4-CF_3_–C_6_H_4_–), as well as the susceptibility of the different microbial strains examined.

**Table 1:** MIC assay of isoxazole–isoxazole 5a–h hybrid compounds^a^

Pdts	Ar	Ar′	Antibacterial activity			Antifungal activity			
			MIC (µmol mL^−1^)						
			*E. coli*	*B. subtilis*	*S. aureus*	*C. albicans*	*A. niger*	*A. flavus*	*F. oxysporum*
5a	4-CH_3_–C_6_H_4_–	C_6_H_5_–	4.878	0.097	NA	0.097	0.156	0.048	0.048
5b	4-CH_3_–C_6_H_4_–	4–CH_3_–C_6_H_4_–	NA	0.094	NA	0.094	0.094	0.094	0.094
5c	4-CH_3_–C_6_H_4_–	4-Cl–C_6_H_4_–	3.289	0.146	NA	0.091	0.091	0.091	0.091
5d	4-CH_3_–C_6_H_4_–	4-CF_3_–C_6_H_4_–	2.582	0.430	NA	0.086	0.086	0.086	0.043
5e	4-Cl–C_6_H_4_–	C_6_H_5_–	4.690	0.938	NA	0.093	0.150	0.093	0.093
5f	4-Cl–C_6_H_4_–	4-CH_3_–C_6_H_4_–	NA	1.828	NA	0.091	0.091	0.091	0.045
5g	4-Cl–C_6_H_4_–	4-Cl–C_6_H_4_–	4.406	1.409	NA	0.088	0.088	0.044	0.088
5h	4-Cl–C_6_H_4_–	4-CF_3_–C_6_H_4_–	4.161	0.083	NA	0.083	0.083	0.083	0.083
Amphotericin B			N.D.	N.D.	N.D.	0.002	N.D.	N.D.	N.D.
Ampicillin			0.0286	0.002	0.108	N.D.	N.D.	N.D.	N.D.
Fluconazole			N.D.	N.D.	N.D.	N.D.	0.130	0.032	0.130

a MIC values represent two independent experiments performed in duplicate. Identical results were obtained in both replicates for all tested compounds; therefore, standard deviation was not applicable.

The analysis of the MICs against *E. coli* reveals notable differences among compounds 5a–h regarding their antibacterial effect. Particularly, compound 5d stands out with an MIC of 2.582 µmol mL^−1^, indicating significant inhibitory activity. Compounds 5c, 5h, and 5g demonstrate slightly enhanced activity, whereas derivatives 5a and 5e, which bear an unsubstituted Ar′ ring, are among the least effective. These trends align with the enhanced activity against *E. coli* when electron-withdrawing groups, like –Cl and –CF_3_, were introduced. The combination of an Ar (4-CH_3_–C_6_H_4_) and a strong electron-withdrawing group in Ar′ (4-CF_3_–C_6_H_4_) is considered to be the most effective, and this combination produced smallest MIC value for 5d. In the case of *B. subtilis*, effective MICs were observed for hybrids 5a–h, where 5h showed high efficacy with an MIC of 0.083 µmol mL^−1^, which is almost equivalent to ampicillin's MIC (0.002 µmol mL^−1^). Compounds 5a–c presented high MIC values of 0.097 and 0.146 µmol mL^−1^, respectively, but compounds 5f and 5g showed loss of efficacy.

Collectively, these results show that the electron-withdrawing groups (–Cl, –CF_3_) enhance the activity of the compounds, particularly against *B. subtilis*, as manifested by compound 5h. None of the prepared compounds were active against *S. aureus*, showing its resistance to this variety of drugs. Although both bacteria are Gram-positive, the compounds showed more activity against *B. subtilis* than *S. aureus* due to the stronger binding of the peptidoglycan layer to teichoic and lipoteichoic acids, with high concentration, in *S. aureus*, thereby decreasing the cell wall permeability of compounds.^59–61^ On the other hand, *B. subtilis* has less compact cell wall architecture, allowing easy access for the compounds, hence a high sensitivity was observed.^62^

Regarding antifungal activity, the compounds 5a–h showed strong activity against *A. niger* and *F. oxysporum*. For *C. albicans*, the compounds 5h, 5g, and 5h had the lowest MICs, and the results were significantly different from the other compounds (*p* < 0.05). Compound 5h showed the strongest activity against *C. albicans*, with an MIC of 0.083 µmol mL^−1^, which was nearly close to that of amphotericin B (MIC = 0.002 µmol mL^−1^). Compounds 5a–c and 5e–f showed MIC values of 0.091 to 0.097 µmol mL^−1^, respectively, indicating strong anti-*C. albicans* activity compared to the reference drug. Analysis of the results shows that the Ar and Ar′ with –CF_3_ and –Cl have a strong to moderate activity against *C. albicans* when substituents are –CH_3_. However, the parent compounds had the lowest antifungal activity. Concerning the antifungal activity against the molds *A. niger*, *A. flavus*, and *F. oxysporum*, all compounds showed potent activity equivalent to that of fluconazole, especially against *F. oxysporum*. For the antifungal activity against *A. niger*, all the compounds with Ar′ having –CF_3_ showed stronger inhibition than fluconazole, specifically compounds 5h, Ar′ (–CF_3_) and 5e, Ar′ (–CF_3_), with MICs of 0.083 µmol mL^−1^, and 0.086 µmol mL^−1^, respectively. Thus, compounds 5b–c and 5f–g showed MICs of 0.088 to 0.094 µmol mL^−1^ lower than that of fluconazole, indicating potent activity.

These results confirm that the substituents Cl and –CF_3_ enhance the antifungal activity. In contrast, fluconazole was more effective than most the compounds against *A. flavus* with exception of 5a and 5g, which had lower MICs of 0.048 and 0.044 µmol mL^−1^, respectively. Against *F. oxysporum*, MIC values of the hybrid series 5a–h ranged from 0.43 to 0.94 µmol mL^−1^ well below fluconazole at 0.130 µmol mL^−1^. It therefore follows that a structural factor of a double isoxazole ring provides importantly high activity against *F. oxysporum* to the tested compounds. Moreover, the substituent groups (–Cl and –CF_3_) make the developed compounds more potent as represented by 5d and 5f.

### 2.3. Antioxidant activity

**Fig. 1:**
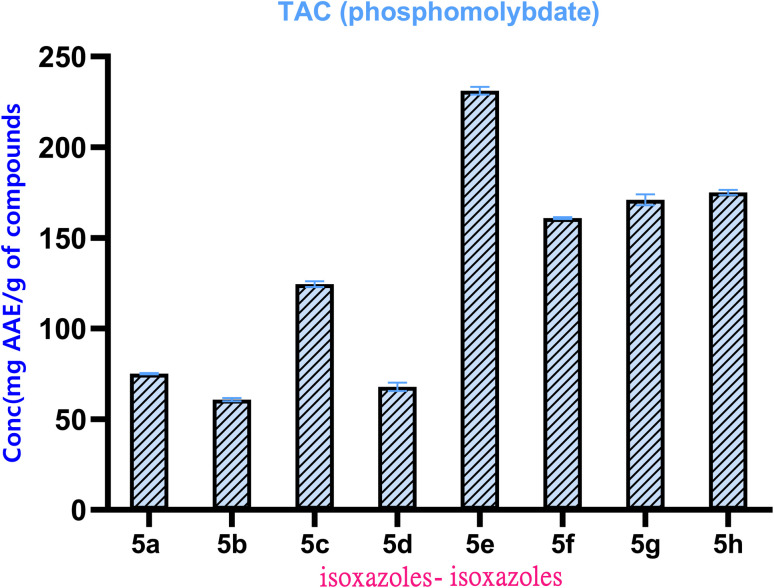
Antioxidant capacity of isoxazole–isoxazole 5a–h hybrid.

The antioxidant capacity of the synthesized hybrids (5a–h) was determined by using the phosphomolybdenum test, as reported in the method of Zengin *et al*.^63^ This test is based on the reduction of Mo(vi) to Mo(v), where a green Mo(v) complex forms, quantifiable by its measurement through a spectrophotometer at a wavelength of 630 nm. This was subjected to calculation, where the result was presented as a milligram of ascorbic acid equivalent to a gram of compound (mg AAE per g). DMSO was used as a negative control, while ascorbic acid was used as a positive control. Replication of the result was done by completing all of the mentioned tasks thrice (Fig. 1).

The results, as shown in Fig. 3, indicate compounds 5e and 5g as the most effective compounds with maximum values (231.20 ± 2.15 and 197.90 ± 3.01 mg AAE per g, respectively), suggesting their high antioxidant capability. Compounds 5e and 5g were significantly different (*p* < 0.05) from the other derivatives. For compounds 5c, 5f and 5h, the reducing activity ranged from 124.51 to 175.06 mg of active principles (mg AAE per g). The lowest activity has been exhibited by compounds 5b (60.83 ± 0.86 mg AAE per g) and 5d (67.93 ± 2.37 mg AAE per g). This significant difference in activity can be explained by the type and position of substituents at the isoxazole ring, which modulate electron density and electron transfer ability. This fully functionalized isoxazole–isoxazole hybrid core structure, in particular with substituent electron donors, appears to be crucial for enhancing the reducing capacity of these compounds.

### 2.4. Structure–activity relationship (SAR)

Structure activity relationship analysis indicates that the substituents present in the aromatic rings (Ar and Ar′) play a critical part in the activity of the 5a–h series of compounds against microorganisms. Generally, the presence of strong electronegative substituents such as –CF_3_ and –Cl groups showed a marked improvement in activity, while the –CH_3_ group showed moderate activity and the least active were the unsubstituted groups. This trend was observed specifically for *E. coli*, among which the most active compound 5d (Ar = 4-CH_3_–C_6_H_4_ and Ar′ = 4-CF_3_–C_6_H_4_) had the lowest MIC value. This suggests that reaching a balance between the electronic and lipophilic properties is critical for the penetration of these molecules into the outer membrane of the Gram-negative bacteria.^64,65^ This trend was also observed with the activity level of the series against *B. subtilis*, where the strongest activity was exhibited by the electron–pull substituents, particulary 5h. This substituent could develop an affinity for the target enzymes for the Gram-positive bacteria.^66,67^ On the other hand, *S. aureus* was resistant to the series of compounds given its thick peptidoglycan cell wall and the presence of permeability barriers and efflux pump.^68,69^ For *C. albicans*, the strongest activity was observed with the 5d, 5g, and 5h series of compounds, thus indicating that electroattractive groups have the ability to improve the binding affinities for the fungal membranes and the biochemical targets.^70,71^ Minimally substituted molecules indicated that higher hydrophobicity and altered electron densities were required for improving the antifungal activity.^72,73^ This trend observed for *C. albicans* was reflected for the mold's series: the derivatives with –CF_3_ and –Cl substituents (5e, 5h, 5d, and 5f) were the least active in terms of MIC and were even more active than fluconazole against *F. oxysporum*. This activity could be due to the two isoxazole nuclei present in the molecule.^41^ This arrangement may be improving the interaction with the cell wall components. This trend indicates the importance of enhancing the antimicrobial activity through the creation of more attractive substituents for this class of compounds. The arrangement for the 5d and 5h series is worth noting for enhancing their potential for other properties.

Analyses based on antioxidant activity show that the level of antioxidant activity potential could be influenced by the nature of the substituent group present in the isoxazole ring structure. Compounds 5e and 5g, containing the –Cl substituent (an electron withdrawing group but also participating in the enhancement of electron density due to delocalization), display high antioxidant activity. However, compounds 5b and 5d with the lowest activity show the significant impact of mesomerism and inductive effects on the relative stabilization of the generated radicals. Moderate activity was noted for compounds 5c, 5f, and 5h, which display less favorable structure characteristics. These observations warrant that the nature and characteristics of the particular substituent group, specifically electron-donating groups, have significant effects in increasing the reducing power of the compounds and can be utilized in designing more active isoxazole derivatives.^74–76^

### 2.5. Molecular docking analysis

To investigate the binding affinity of compounds 5a–h with the desired bacterial (1KZN and 5TW8) and fungal (1EAG and 3DJE) protein targets that are of prime importance in the pathogenicity of microbes. The docking study revealed a high binding affinity with considerable dock scores of Δ*G* (Gibbs free energy) in the range of −8.3 kcal mol^−1^ to −11.8 kcal mol^−1^ (Table 2). Among the tested compounds 5a, 5b and 5c proved to have elite dual antibacterial and antifungal properties with high binding energies of up to −11.7 kcal mol^−1^ for the fungal protein 3DJE and greater than −9.0 kcal mol^−1^ for the bacterial protein targets. Compound 5g proved to have the highest binding energy of −11.8 kcal mol^−1^ for the fungal protein and also retains high antibacterial properties. The compound possesses the ability to have broad-spectrum activity. Also, the binding energy of the newly designed compounds proved to be much higher than the standard antibacterial and antifungal compounds ampicillin (−7.3 kcal mol^−1^), fluconazole (−6.6 kcal mol^−1^ to −7.9 kcal mol^−1^), and amphotericin B (−8.1 kcal mol^−1^). The result indicated better binding of the newly synthesized compounds with the active site of the microbial protein. It appears that the enhanced docking ability of these compounds provides a robust basis for being capable of blocking the crucial enzymatic function necessary for survival, and hence, they are promising leads for the development of new drugs targeting the pathogen. However, further validation and study are necessary to establish biological activity.

**Table 2:** Docking scores of leading compounds and reference drugs against key proteins involved in antibacterial and antifungal activities

Compounds	Antibacterial target		Antifungal target	
	1kzn	5tw8	1eag	3dje
5a	−9.8	−9.9	−10.1	−11.6
5b	−9.1	−9.4	−8.5	−11.7
5c	−9.0	−9.3	−8.5	−11.7
5d	−9.6	−9.3	−9.5	−8.3
5e	−9.4	−9.6	−9.4	−11.4
5f	−8.7	−10.2	−10.5	−8.5
5g	−9.0	−10.3	−10.0	−11.8
5h	−9.2	−10.2	−10.6	−8.8

**Scoring for the reference drug (kcal mol^−^** ^**1**^ **)**				
Amphotericin B	***	−8.1	***	***
Ampicillin	−7.3	***	***	***
Fluconazole	***	***	−6.6	−7.9

#### 2.5.1. Protein–ligand interaction analysis

**Fig. 2:**
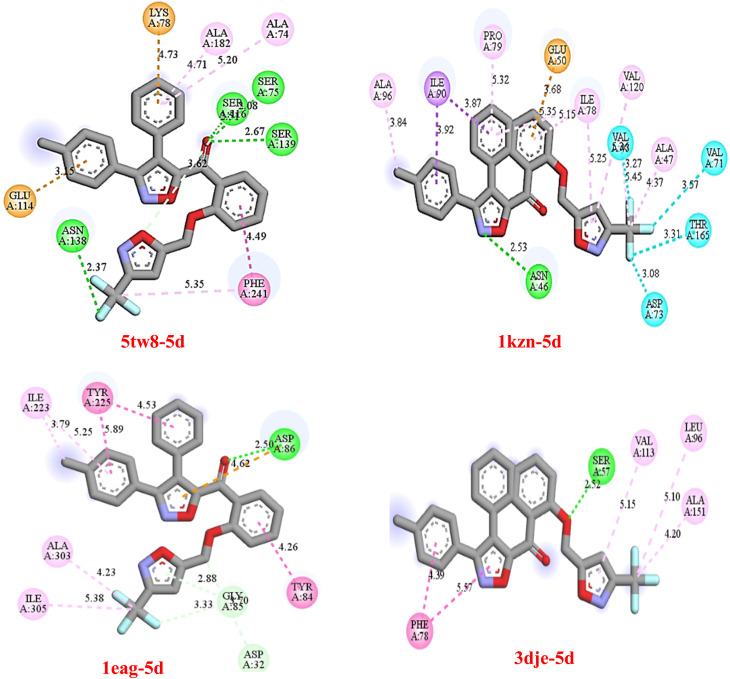
2D visualization of key ligand 5d's interactions with active sites on target proteins using Discovery Studio.

A thorough study on the interactions of proteins and ligands was done to determine the binding affinities of target proteins with the most energetic and bioactive ligands. A study done using Discovery Studio generated 2D diagrams showing the involvement of key residues within the binding sites of proteins and ligands in interactions such as hydrophobic interactions, hydrogen bonding, and electrostatic interactions (Fig. 2).

### 2.6. Computational ADME-Tox and drug-likeness analyses

For the assessment of the pharmacokinetic behavior and drug likeness of the identified molecules, an in-depth ADME-Tox study was conducted. The Swiss ADME and pkCSM online servers were employed to predict the parameters of interest, including oral bioavailability, solubility, lipophilicity, and toxicity. Table 3 represents the bioavailability characteristics of the eight most active molecules selected based on the data obtained from the docking simulation studies.

**Table 3:** Predicted bioavailability of the compounds 5a–h

Molecule	5a	5b	5c	5d	5e	5f	5g	5h
MW (size)	512.55	450.49	470.90	504.46	532.97	547.00	567.42	600.97
GI absorption	Low	High	Low	Low	Low	Low	Low	Low
Fraction Csp^3^ (saturation index)	0.06	0.11	0.07	0.11	0.03	0.06	0.03	0.06
#Rotatable bonds (flexibility)	8	7	7	8	8	8	8	9
#H-bond acceptors	6	6	6	9	6	6	6	9
#H-bond donors	0	0	0	0	0	0	0	0
TPSA (polarity)	78.36	78.36	78.36	78.36	78.36	78.36	78.36	78.36
XLOGP3 (lipophilicity)	7.31	6.05	6.61	6.57	7.57	7.93	8.20	8.45
MLOGP	3.76	3.01	3.28	3.57	4.03	4.21	4.48	4.75
ESOL log *S* (insolubility)	−7.74	−6.59	−7.07	−7.14	−8.03	−8.33	−8.62	−8.88
Lipinski #violations	1	0	0	1	1	2	2	2
Bioavailability score	0.55	0.55	0.55	0.55	0.55	0.17	0.17	0.17
PAINS #alerts	0	0	0	0	0	0	0	0
Synthetic accessibility	4.41	4.13	4.03	4.18	4.29	4.41	4.29	4.40

Compounds 5a–h were subjected to *in silico* ADME-Tox analyses utilizing the SwissADME and pkCSM platforms to provide preliminary insights into their pharmacokinetic and drug-like characteristics. Table 3 summarizes the expected physicochemical properties and bioavailability characteristics, while Table 4 presents the full ADME-Tox profiles. The results show that numerous compounds surpass the acceptable molecular weight threshold of 500 g mol^−1^ and have high lipophilicity values (XLOGP3 > 5), resulting in Lipinski rule breaches. These physicochemical properties may have a deleterious impact on water solubility and oral bioavailability. The estimated ESOL log *S* values (−6.59 to −8.88) indicate low water solubility across the sequence, especially for compounds 5f–5h (Table 3). Among the compounds tested, compound 5d had a substantially better projected safety profile, owing to the absence of hepatotoxicity alerts, AMES toxicity, hERG I inhibition, and skin sensitization, as shown in Table 4. It also had a high projected intestinal absorption rate (95.49%) and acceptable Caco-2 permeability (1.089) (Table 4). However, its molecular weight (504.46 g mol^−1^) and lipophilicity (XLOGP3 = 6.57) are slightly higher than the ideal range suggested by Lipinski's rule of five (Table 3), indicating that structural optimization may be required to improve its physicochemical balance. The remaining analogues (5a, 5c, 5e–5h) had moderate to low predicted GI absorption and greater lipophilicity values, which could account for their lower bioavailability ratings (Table 3). Furthermore, some substances were predicted to inhibit CYP2C9 and CYP3A4 (Table 4), highlighting the possibility of drug–drug interactions. The total clearance and distribution parameters presented in Table 4 indicate moderate elimination rates and little central nervous system penetration throughout the series. It is important to emphasize that these findings are based exclusively on computational predictions and should be interpreted as preliminary screening data rather than definitive evidence of drug potential. Experimental validation through *in vitro* and in *vivo* pharmacokinetic and toxicity studies is required to confirm these theoretical assessments.

**Table 4:** ADME-Tox profiles of the compounds 5a–h

ADMET properties		5a	5b	5c	5d	5e	5f	5g	5h
Absorption	Water solubility (log mol L^−1^)	−2.933	−3.276	−3.525	−3.460	−2.933	−3.072	−3.069	−3.063
	Caco2 permeability (log Papp)	1.05	1.10	1.02	1.09	1.04	1.07	1.06	1.11
	Intestinal absorption (%)	96.85	100.0	97.17	95.49	95.39	97.73	96.27	94.89
	Skin permeability (log Kp)	−2.735	−2.735	−2.735	−2.735	−2.735	−2.735	−2.735	−2.735
Distribution	VDss (log L kg^−1^)	−0.487	−0.335	−0.284	−0.214	−0.484	−0.395	−0.390	−0.390
	Fraction unbound	0.383	0.363	0.331	0.333	0.383	0.365	0.365	0.369
	BBB permeability (log BB)	−0.868	−0.866	−0.984	−1.245	−1.043	−1.098	−1.274	−1.546
	CNS permeability (log PS)	−1.596	−1.882	−1.810	−1.760	−1.556	−1.430	−1.390	−1.349
Metabolism	CYP2D6 substrate	No	No	No	No	No	No	No	No
	CYP3A4 substrate	Yes	Yes	Yes	Yes	Yes	Yes	Yes	Yes
	CYP1A2 inhibitor	No	No	No	No	No	No	No	No
	CYP2C19 inhibitor	No	No	No	No	No	No	No	No
	CYP2C9 inhibitor	Yes	Yes	Yes	Yes	Yes	Yes	Yes	Yes
	CYP2D6 inhibitor	No	No	No	No	No	No	No	No
	CYP3A4 inhibitor	Yes	Yes	Yes	Yes	Yes	No	No	No
Excretion	Total clearance (log mL min^−1^ kg^−1^)	0.483	0.388	0.238	0.132	0.259	0.201	0.127	0.180
	Renal OCT2 substrate	No	No	No	No	No	No	No	No
Toxicity	AMES toxicity	No	No	No	No	No	No	No	No
	hERG I inhibitor	No	No	No	No	No	No	No	No
	Hepatotoxicity	Yes	Yes	Yes	No	Yes	Yes	Yes	Yes
	Skin sensitization	No	No	No	No	No	No	No	No

### 2.7. Molecular dynamic simulation analysis

#### 2.7.1. Root-mean-square deviation (RMSD) analysis

**Fig. 3:**
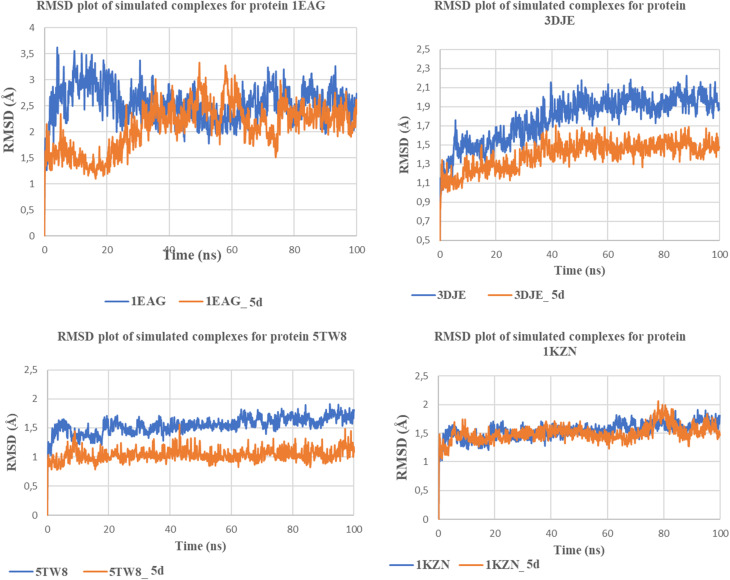
RMSD profiles of protein–ligand complexes compared to apo proteins during 100 ns md simulations.

Ligand binding positions are revealed as a static system in molecular docking studies. To overcome these constraints, MDS simulation was performed for 100 ns on the 5d ligand in complex with critical bacterial protein targets (1KZN, 5TW8) and fungal proteins (1EAG, 3DJE). The RMSD graph was used for interpreting the stability of the protein or ligand structure at the binding site depicted in Fig. 3. In the 1EAG:5d complex, it was observed that the protein shows small fluctuations at the initial stages, finally becoming stable at 2 Å after 30 ns, whereas the apoprotein shows higher fluctuations at approximately 4 Å; these observations confirm that the presence of 5d increases the stability of the protein structure, as it remains strongly bound at the active site. Likewise, for the 3DJE:5d complex, the protein structure shows stability at approximately 1.5 Å after an equilibration time of 25 ns, whereas higher fluctuations are observed for the apoprotein at values below 2.5 Å, making it evident that 5d helps maintain binding stability at the active site. A similar trend was observed for the 5TW8:5d complex, where the protein structure becomes stable at 1 Å. On the other hand, the apoprotein showed variation up to 1.9 Å, which revealed less structural changes and strengthened the retention of the ligand inside the pocket. Finally, the 1KZN-5d complex showed that the RMSD values of the protein and the ligand were approximately 2 Å and less than 1.9 Å, respectively, over the entire simulation time of 100 ns, further justifying the ligand's stability. For all systems, the RMSD values of the ligand–protein complexes were consistently smaller than that of the free protein, which showed, at times, slight increases, emphasizing the stabilizing impact of the binding of 5d. The overall data indicate that the 5d binds to the active pockets of each target molecule without inducing any structural changes. The synchronized RMSD values of both the ligand and the protein further reinforce the integrity of the interactions between 5d and the targets, which establish the efficacy of 5d as a dual antibacterial and antifungal agent.

#### 2.7.2. Root-mean-square fluctuation (RMSF) analysis

**Fig. 4:**
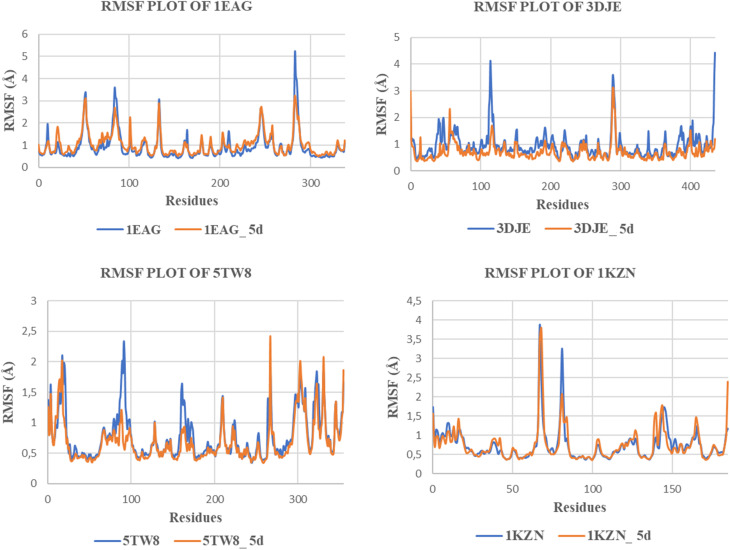
RMSF profiles of protein–ligand complexes compared to apo proteins.

RMSF (Root Mean Square Fluctuation) analysis is used to observe residue-level dynamics in protein–ligand complexes in comparison to their apo forms (Fig. 4). Generally, the values of residue-level relaxation in the form of RMSF values in the protein systems simulated were in the normal range of 0.5 to 2.5 Å values, which depict stability in the systems apart from loops that were flexible. For the 1EAG-5d complex system, the RMSF pattern almost completely overlapped that of apo 1EAG, indicating slight decreases in flexibility in various regions of loops 1 to 5 when complexed to 5d. For the 3DJE-5d complex system, the values were relatively low, ranging between 1 to 2 Å in most regions apart from where residue 120 exhibited slight maxima. Likewise, in the case of the 1KZN-5d complex, there were observed to be low RMSF values throughout the protein chain with minimal deviations from the apo protein structures, as in all cases, it has been noted that binding by 5d maintained the stability of the protein in its native form. Likewise, in the 5TW8-5d protein complex, it has been observed that its RMSF values are highly stable with minimal deviations with values mostly being less than 2 Å with only minor peaks at around residues 80–100, which is natural and is not due to perturbation from binding with any particular compound.

#### 2.7.3. Protein–ligand interaction analysis of selected complexes

**Fig. 5:**
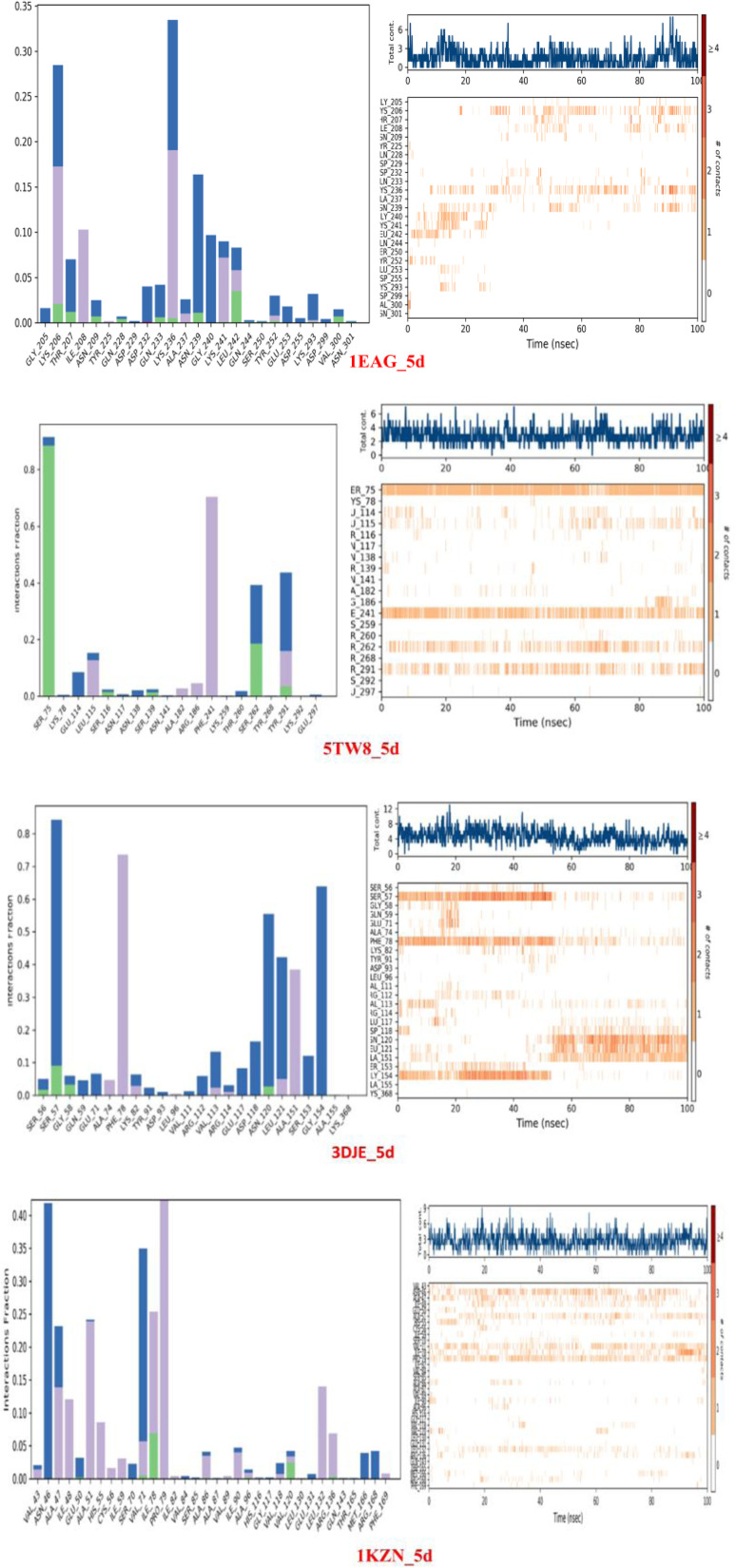
Histogram of protein–ligand interactions and corresponding residue contact numbers for the simulated complexes.

Protein–ligand interaction analysis of complexes with bacterial 1KZN, 5TW8, and fungal 1EAG, 3DJE targets in a 100 ns molecular dynamics simulation showed that both adopted stable and well-defined binding modes along the trajectory, sustained by persistent hydrogen bonding, hydrophobic contacts, and water-mediated bridges. In the 1EAG complex, residues like GLU117, ASP120, and TYR168 had high interaction fractions >0.8 that are consistently stabilizing the ligand along the trajectory. Further contacts from ASN172 and HIS215 contribute to ligand anchoring, reflecting a robust binding profile. The 3DJE complex showed equally high interaction levels with consistent engagement of the residues GLU141, TYR144, and PHE201, aided by occasional hydrogen bond engagement with the residues SER199 and GLU223. The regular high level of engagement of these residues above 0.7 reiterates the importance of these residues for the stability of the ligand. In the bacterial target protein 1KZN, the ligand showed consistent engagement with the residues ASP45, GLU73, and LYS97, aided by moderate regular engagement with the residues VAL100 and THR103. For the 5TW8 structure, the ligand showed consistent engagement with the hydrophobic/polar residues MET56, TYR60, and ASP88, with regular hydrogen bond engagement with the residue GLU91. The ligand showed consistent engagement for almost all the selected residues with interaction fractions close to the maximum of 1.0 throughout the run. The ligand engagement heatmaps reveal consistent engagement of the ligand with these residues, while the number of engagement plots showed non-deviating engagement with consistent ligand engagement throughout the run. Overall, the above findings demonstrate the ligand's stability across the bacterial and fungal proteins with diverse structural characteristics, establishing it further as an effective broad-spectrum antimicrobial agent (Fig. 5).

## 3. Materials and methods

### 3.1. Chemistry

The supplementary file includes a detailed description of reagents and solvents for the synthesis of target isoxazoles, instrumentation for analysis of characterization spectra, and syntheses.

### 3.2. Antimicrobial activity

The supplementary file also holds information about the methodological approach in the bacterial and fungal strains that will be used to measure the antimicrobial potential of compounds 5a–h.

### 3.3. Antioxidant activity

The procedure used for screening the antioxidant activity of compounds 5a–h is given in SI.

### 3.4. Molecular docking simulation

For the exploration of the antimicrobial properties of selected natural bioactive compounds, molecular docking simulation studies were also performed with the help of AutoDock Vina. The major aim was to assess and study the binding affinity of these natural bioactive molecules with selected microbial target proteins.^77^ For this purpose, two antibacterial target proteins – DNA gyrase subunit B (PDB ID: 1KZN), which takes part in bacterial DNA synthesis, and penicillin-binding protein 2a (PDB ID: 5TW8), which plays an imperative function in cell wall synthesis, as well as two antifungal target proteins 1EAG, linked with *Candida albicans*, and 3DJE, obtained from *Aspergillus fumigatus*, which performs a pivotal function in fungal pathogenicity and life,^77^ were selected.^78^

### 3.5. Protein structure preparation

High-resolution crystal structures of the target microbial proteins were sourced from the RCSB Protein Data Bank (https://www.rcsb.org/) in PDB format. These proteins consisted of both antibacterial and antifungal targets that are associated with microbial pathogenesis. Structure visualization was done using BIOVIA Discovery Studio, with structure preparation using AutoDock Tools version 4.2.6. The preparation steps included the removal of water molecules and heteroatoms, the addition of polar hydrogen atoms, and the assignment of partial charges, which included Kollman charges in the case of proteins and Gasteiger charges in the case of ligands. The optimized protein structures were then saved in PDBQT format to make them compatible with the AutoDock Vina tool and to increase the accuracy of the docking simulations, as pointed out in ref. 79. To accurately target the active sites of the proteins and ensure that the complete active site was covered in simulating protein–ligand interactions, the docking boxes were placed around the coordinates and sizes of the 3D grid boxes as presented in Table 5.

**Table 5:** Coordinates of the centers and dimensions of the 3D docking grid boxes

PDB ID	Target	Centers (Å)			Size (Å)	Exhaustiveness
		*X*	*Y*	*Z*	*X*, *Y*, *Z*	=
1kzn	Antimicrobial	19.1	30.3	34.7	20	9
5tw8	Antimicrobial	21.3	62.2	36.1	40	9
1eag	Antifungal	41.6	24.9	13.6	40	9
3dje	Antifungal	43.2	7.7	39.1	40	9

### 3.6. Ligand preparation

The identified bioactive molecules were initially drawn and optimized with the aid of Chem3D software in addition to energy minimization with Avogadro software. The molecules' geometry optimization was achieved through the steepest descent algorithm with the MMFF94 energetic term to obtain conformers with less energy as well as optimized conformers ready for docking experiments.^80^ Energy-optimized molecules' structures were then transformed to PDBQT files ready to be evaluated by AutoDock Vina to predict binding affinity to targeted molecules in agreement with previous docking experiments.^79^ Finally, a comparative study between the binding affinity scores from docking experiments and those from known available medications with well-known effectiveness as antibacterial and antifungal agents provided a relative effectiveness level of all test molecules *in vitro*.

### 3.7. Computational ADME and drug-likeness analysis

In order to make an assessment about the pharmacokinetic optimality and potency of these compounds, an extensive *in silico* screening for their ADME-Tox profiles was carried out. Based on their SMILES codes, the https://www.swissadme.ch server, available as an open-access WEB resource, was used for these compounds to calculate their physicochemical and pharmacokinetic characteristics such as lipophilicity, water solubility, gastrointestinal absorption, and their aptness for the rule of five, which are essential criteria for analyzing their oral bioavailability and drug likeness.^81^ At the same time, another program named pkCSM was used for an extensive analysis relating to their ADME-Tox, particularly for their hepatotoxic, skin sensibility, blood–brain barrier permeability, and cytochrome P450 binding properties, as an attempt to get an insight into their pharmacokinetic optimality. In this way, an extensive pharmacokinetic screening was performed, aiming at the early selection of these compounds with potentially favorable pharmacokinetic profiles and low toxicity. These profiles work as filters at the early stages prior to their further laboratory tests.^82^

### 3.8. Molecular dynamics simulation (MDS)

Molecular dynamics simulation (MDS) was employed to explore the dynamic behavior and stability of the most promising ligand–protein complex at the atomic level. All simulations were conducted using Desmond, a component of the Schrödinger suite, over a 100 ns-time frame. The protein structure was first prepared using Schrödinger's Protein Preparation Wizard at a physiological pH of 7.4 to ensure structural accuracy and stability.^83^ The solvated system was constructed *via* the System Builder module using the TIP3P water model, with system neutrality achieved by the addition of Na^+^/Cl^−^ counterions at a 0.15 M salt concentration. The OPLS3e force field was applied to define atomic interactions. Equilibration began under the NVT ensemble with gradual temperature increase, followed by the NPT ensemble regulated by the Nose–Hoover thermostat (300 K) and the Martyna–Tobias–Klein barostat (1.01325 bar). During the 100 ns production run, key parameters such as root mean square deviation (RMSD), root mean square fluctuation (RMSF), and protein–ligand interaction profiles were monitored to assess the conformational stability and binding performance of the complex.^84^

Molecular dynamics simulation (MDS) was used to investigate the dynamic properties and stability of the most favorable ligand–protein complex at the atomic level. All MDS runs were carried out by the Desmond module of the Schrödinger software suite over a 100 ns simulation time course. Prior to the simulation, the protein was prepared using the Protein Preparation Wizard module of the Schrödinger suite at physiological pH 7.4 to guarantee structural correctness and conformational stability.^83^ The solvent system was set up using the System Builder module with the TIP3P water force field; neutrality was ensured by the addition of Na^+^/Cl^−^ counter-ions at 0.15 M concentration 83. The OPLS3e force filed was used to describe the atomic interactions. The equilibration procedure was performed separately at first in the NVT ensemble with linear temperature scaling, followed by the NPT ensemble with control by the Nose–Hoover temperature regulator at 300 K and the Martyna–Tobias–Klein pressure regulator at 1.01325 bar 83. During the 100 ns simulation run, respect parameters like the root mean squared deviation (RMSD), the root mean squared fluctuation (RMSF), or protein–ligand interaction analyses were applied to examine the conformational and stabilizing properties of the ligand–protein complex.^84^

## 4. Conclusions

The current research works highlight the effective synthesis of a new series of isoxazole–isoxazole hybrids (5a–h) successfully isolated with substantial yields, where a comprehensive characterization is presented employing FT-IR, NMR (^1^H, ^13^C, 2D), and HR-MS. Evaluation of the numerous biological properties taken into account revealed that the hybrids presented potent and variable activities. They showed inhibitory effect towards different bacteria, including Gram-positive, Gram-negative, in addition to some fungal, yeast, and mold strains. Compounds 5d and 5h demonstrated substantial activity against *E. coli*, in addition to *B. subtilis*, while 5h, 5g presented pronounced activity towards *C. albicans*, *A. niger*, *A. flavus*, in addition to *F. oxysporum*. Notably, the hybrids presented exhibited improved activity compared to fluconazole, the reference drug. Also, in conjunction, evaluation of the antioxidant activity demonstrates that 5e, 5g presented substantial reducing capability. Importantly, the current works highlight the pivotal impact of the synthesized substituents in modifying the biological activities of the new isoxazole hybrids.

*In silico* validation was done to confirm these findings. Using molecular docking and molecular dynamics simulations, it was proven that the isoxazole–isoxazole compounds, especially 5d, have stable and various interactions with bacterial (1KZN, 5TW8) and fungal (1EAG, 3DJE) protein structures using mechanisms such as hydrogen bonding, hydrophobic interactions, and water bridging. Within this group of compounds, the binding affinity of 5d stands out as the strongest. By the root mean square fluctuation (RMSF) calculation, these interactions help in decreasing the fluctuation of residues and thus can enhance the binding affinity of the complex. The ADME-Tox profile of the *in silico* analysis suggests that the most promising candidate with high gastrointestinal absorption, drug-likeness, and balanced pharmacokinetic properties suitable for development as an antimicrobial agent is 5d.

It is pertinent to highlight that the significance of the isoxazole–isoxazole ring structure is made explicit in this research work. It is clear from the above-stated observations that the outcomes also verify the efficiency and effectiveness associated with the isoxazole–isoxazole ring structure. It can thus be added that the feasibility associated with the experimentally designed synthesis contributes effectively towards validating the efficiency associated with the isoxazole–isoxazole framework.

## Conflicts of interest

The authors have no conflicts of interest to declare that are relevant to the content of this article.

## Supplementary Material

RA-016-D6RA01005A-s001

## Data Availability

All data generated or analysed during this study are included in the article and its supplementary information (SI). Supplementary information: spectroscopic data and copies of IR, NMR and HRMS spectra of the newly synthesized compounds, and all additional details. See DOI: https://doi.org/10.1039/d6ra01005a.
