# Supplementary material for: New isoxazole-based heterocyclic hybrids with dual antimicrobial and antioxidant bioactivity: integrated synthesis, in vitro assessment, and computational exploration
Source: RSC Adv. 2026 Mar 10;16(15):13515–28. doi: 10.1039/d6ra01005a (PMC12973378; doi:10.1039/d6ra01005a)
Supplement: RA-016-D6RA01005A-s001 [file RA-016-D6RA01005A-s001.pdf]

Supplementary Information (SI) for RSC Advances.
This journal is © The Royal Society of Chemistry 2026

Supplementary information for paper

New Isoxazole-Based Heterocyclic Hybrids with Dual Antimicrobial and Antioxidant Bioactivity: Integrated Synthesis, *In Vitro* Assessment, and Computational Exploration

Aziz ARZINE[a], Soumia AIT ASSOU[b], Lamiae ELBOUAMRI[c], Mohammed CHALKHA[*,d,a], Asmae NAKKABI[d,a], Samir CHTITA[c], Reem M. ALJOWAIE[e], Mourad A. M. ABOUL-SOUD[*,F], Mohammed El HASSOUNI[b], John P. GIESY[g,h,i], Mohamed El YAZIDI[a]

[a]Engineering Laboratory of Organometallic, Molecular Materials and Environment, Faculty of Sciences Dhar EL Mahraz, Sidi Mohamed Ben Abdellah University, P.O. Box 1796 (Atlas), 30000 Fez, Morocco

[b]Biotechnology, Environment, Agri-Food and Health Laboratory, Faculty of Sciences Dhar El Mahraz, Sidi Mohamed Ben Abdellah University, BP. 1796, Atlas, Fez, Morocco

[c]Laboratory of Analytical and Molecular Chemistry, Faculty of Sciences Ben M'Sik, Hassan II University of Casablanca, Casablanca B.P 7955, Morocco

[d]Laboratory of Materials Engineering for the Environment and Natural Resources, Faculty of Sciences and Techniques, University of Moulay Ismail of Meknes, B.P 509, Boutalamine, 52000, Errachidia, Morocco

[e]Department of Botany and Microbiology, College of Science, King Saud University, P.O. Box 2455, Riyadh 11451, Saudi Arabia;

[f]Center of Excellence in Biotechnology Research, College of Applied Medical Sciences, King Saud University, Riyadh 11433, Saudi Arabia;

[g]Department of Veterinary Biomedical Sciences and Toxicology Centre, Western College of Veterinary Medicine, University of Saskatchewan, Saskatoon, SK S7N 5B4, Canada

[h]Department of Integrative Biology and Center for Integrative Toxicology, Michigan State University, East Lansing, MI 48824, USA;

[i]Department of Environmental Sciences, Baylor University, Waco, 76706, USA

***Corresponding authors**: mohammed.chalkha1@usmba.ac.ma (Mohammed CHALKHA), maboulsoud@ksu.edu.sa (Mourad A. M. ABOUL-SOUD)

Table of contents

1. **General information** **3**

2. **Synthesis methods** **3**

   2.1 **Synthesis of 3-aryl-5-(2-hydroxybenzoyl)-4-phenylisoxazoles 3a-b** 3

   2.2 **Synthesis of isoxazoles 4a-b** 4

   2.3 **Synthesis procedure for 5a-h hybrids** 4

3. **Experimental Protocol for the Evaluation of Antimicrobial and Antioxidant Activities of Compounds 5a–h** **4**

   3.1 **Determination of minimum inhibitory concentration (MIC)** 4

   3.2 ***In vitro*, antioxidant test** 5

4. **Characterization data** **5**

   4.1 **Spectroscopic data of the synthesized hybrids 5a-h** 5

   4.1 **Copies of the (1H, 13C) NMR, IR and HRMS spectra of all new hybrid compounds (5a-h)** 11

References: **32**

1. General information

All reagents and solvents were of A. R. grade. They were purchased from Sigma-Aldrich and other commercial suppliers and were used without further purification. The progress of the reactions was monitored by TLC, performed on pre-coated Merck silica gel 60 F_{254} plates. Column chromatography was carried out using Merck silica gel (70-230 mesh) and eluting with n-hexane/ether solutions. Melting points were determined using a KOFLER Bench apparatus. ^{1}H and ^{13}C NMR spectra were recorded at room temperature on a BRUKER AVANCE II 300 Ultra-Shield (300 MHz for ^{1}H and 75 MHz for ^{13}C) spectrometer using $CDCl_3$ and DMSO-d_6 solvents. The Jmod experiment, which was employed for the ^{13}C NMR spectra, reveals information on the number of ^{13}C signals (CH_3, CH_2, CH and Cq). CH_3 and CH carbons are represented by negative signals in these spectra, while CH_2 and C quaternary carbons are represented by positive signals. ^{1}H and ^{13}C NMR chemical shifts (δ) are expressed in ppm referenced to the residual proton signals in the solvent used, and proton-proton coupling constants (J) are reported in Hz. The spin multiplicities are reported as singlet (s), doublet (d), triplet (t), multiplet (m), doublet of doublets (dd), doublet of quadruplet (dq), triplet of doublets (td), doublet of triplets (dt) and broad (br). The IR spectra were recorded in the range of 450–4000 cm^{-1}on a BRUKER VERTEX 70 FT-IR Spectrometer, and peak positions are given in cm^{-1}. The high-resolution mass spectra were recorded using the Exactive Plus quadrupole orbitrap mass spectrometer (Thermo), equipped with an Electrospray ionization (ESI) source operating in a positive and negative ion mode. Aurone **1** used as starting material in this work was synthesized following the procedures described earlier [1,2]. The synthesis of **3a-b** and their characterization data are described in our previously published article [3,4].

2. Synthesis methods

2.1 Synthesis of 3-aryl-5-(2-hydroxybenzoyl)-4-phenylisoxazoles 3a-b

In a 100 mL flask, 1 mmol of (*Z*)-2-benzylidenebenzofuran-3(2*H*)-one and 1.2 mmol of the appropriate hydroxamoyl chloride were dissolved in 20 mL of chloroform and 1.2 mmol triethylamine was added. The mixture was stirred at room temperature, and the progress of the reaction was monitored by TLC. After consumption of the reagents, the solvent was removed to provide a crude product. The residue was crystallized in ethanol and the precipitate was isolated by filtration and washed with cold ethanol.

2.2 Synthesis of isoxazoles 4a-b

In a 100 mL flask, 1 mmol of 3-aryl-5-(2-hydroxybenzoyl)-4-phenylisoxazole **3** and potassium carbonate (K_2CO_3) were dissolved in 20 mL of DMF and the mixture was stirred at room temperature for 5 minutes before adding 1.1 mmol of propargyl bromide. The reaction was stirred magnetically until the reagents were consumed, as monitored by TLC. After the reaction was complete, the solvent was removed under reduced pressure and the resulting residue was diluted with dichloromethane (DCM). The mixture was washed with water and the organic layer was dried over anhydrous sodium sulfate (Na_2SO_4), filtered, and concentrated. The crude product was then purified by silica gel column chromatography using a hexane/diethyl ether (4/1) mixture as the eluent, yielding alkylated isoxazoles **4a-b** in good yield[5].

2.3 Synthesis procedure for 5a-h hybrids

In a 100-mL flask, 1 mmol of *o*-propargylated isoxazoles and 1.2 mmol of arylhydroxamoyl chlorides are dissolved in 40 mL of chloroform. Subsequently, 1.2 mmol of anhydrous triethylamine is added dropwise to this mixture. Subsequent to the addition of the reagents, the reaction mixture is subjected to magnetic stirring at room temperature for a predetermined duration. The reaction is meticulously monitored by TLC, and upon the confirmation of its conclusion, the reaction mixture is meticulously transferred to a separating funnel and thoroughly washed three times with water. Subsequently, the organic phase undergoes a drying process over anhydrous sodium sulfate (Na_2SO_4), followed by filtration to remove the solvent. The residue obtained is then purified by column chromatography with hexane/ether (3/2) as the eluent. The fractions obtained are recrystallized in ethanol.

3. Experimental Protocol for the Evaluation of Antimicrobial and Antioxidant Activities of Compounds 5a–h

3.1 Determination of minimum inhibitory concentration (MIC)

The MIC value was evaluated using the microdilution method, following the guidelines of [6,7] and the protocol adopted by [8]. In a 96-well microplate, each well contained the culture medium, appropriate test concentration, and approximately 10^3 cells/spores of the yeast/mold or 10^5 of the tested bacteria (*E. coli* and *B. subtilis*). Stock solutions have been made of **5a-h** with the following concentrations: 25000, 20000, 18000, 15000, 12000, 10000, 8000, 5000, 2500, 1000, 800, 500,

250, 100, 80 and 50 µg/ml. 10 µL of stock solution shall be added to each well to achieve the required concentration; 2500, 2000, 1800, 1500, 1200, 1000, 800, 500, 250, 100, 80, 50, 25, 10, 8 and 5 µg/ml, followed by the inoculum. The well 11, which contained the culture medium plus the inoculum, and the well 12, including only the culture medium, represented the positive and negative growth controls, respectively. Reference antimicrobial agents were included as assay controls: ampicillin and amphotericin B (concentration range: 1–10 µg/mL) and fluconazole (concentration range: 1–40 µg/mL). The microplates were then incubated at 30°C for C. albicans, 37°C for bacteria, and 25°C for the molds Aspergillus niger, Aspergillus flavus, and Fusarium oxysporum, respectively, for 24 to 48 h. Following incubation, for each well, 20 µL of a 0.01 % of the triphenyl tetrazolium chloride solution were added and the microplate was re-incubated at 30°C for an additional 3 h to evaluate the results. The growth of microbial strains was indicated with a pink color, and the MIC value was assessed as the smallest concentration of compound that did not cause the pink color. MIC assay was assessed in two independent replicates under identical concentrations and conditions.

3.2 *In vitro*, antioxidant test

The total antioxidant activity of the **5a-h** compounds under study was also evaluated using the phosphomolybdenum method according to the procedure described by Zengin et *al.* [9]. A volume of 25 µL of each compound or ascorbic acid (utilized as a standard) was amalgamated with 1 mL of reagent solution (comprising 6 M sulfuric acid, 28 mM sodium phosphate, and 4 mM ammonium molybdate). The tubes were then subjected to an incubation process in a water bath maintained at a temperature of 95°C for a duration of 90 minutes. Subsequent to cooling the tubes to ambient temperature, the spectrophotometer was utilized to measure the relative light absorption at 695 nanometers (nm) in relation to a blank sample. This procedure was executed using a PerkinElmer Lambda 40 UV/VIS spectrophotometer. The antioxidant capacity of compounds **5a-h** was evaluated in ascorbic acid equivalents (mg AA/g compound).

4. Characterization data

4.1 Spectroscopic data of the synthesized hybrids 5a-h

(4-phenyl-3-(p-tolyl)isoxazol-5-yl)(2-((3-phenylisoxazol-5-yl)methoxy)phenyl)methanone **(5a) : White solid**; **Yield** = 70%; **M.p.**: 204-206 °C.

IR (KBr, νmax, cm^{-1}): 3110 (C4–H), 3042 (Ar–H), 2922, 2868 (C–H_{aliph}), 1669 (C=O), 1597 (C=N).

^{1}H NMR (300 MHz, $CDCl_3$) (δ in ppm): 2.30 (s, 3H, CH_3), 5.09 (s, 2H, CH_2), 6.43 (s, 1H, CH), 6.90 (d, 1H, J = 6 Hz, Ar–H), 6.98 (d, 2H, J = 6 Hz, Ar–H), 7.10–7.53 (m, 12H, Ar–H), 7.62–7.69 (m, 3H, Ar–H).

^{13}C NMR (75 MHz, $CDCl_3$) (δ in ppm): 21.32 (CH_3), 61.90 (O–CH_2), 101.29 (CH), 112.16, 121.91, 122.02, 124.83, 126.82, 127.71, 128.23, 128.27, 128.41, 128.49, 128.81, 129.20, 129.99, 130.06, 130.84, 134.27, 139.77, 156.58, 162.08, 162.47, 163.02, 167.27, 183.48 (C=O).

HRMS (ESI, m/z): calculated mass for $[C_{33}H_{24}N_2O_4 + H]^+$ = 513.17433; found mass = 513.16913, calculated mass for $[C_{33}H_{24}N_2O_4 + Na]^+$ = 535.16283; found mass = 535.15033.

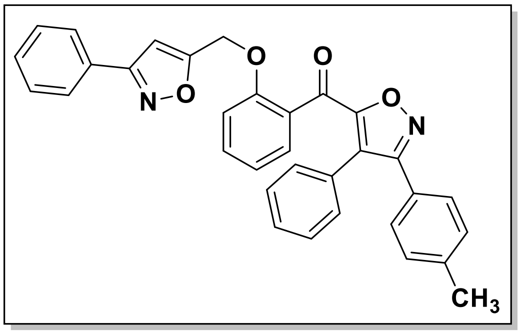

***(4-phenyl-3-(p-tolyl)isoxazol-5-yl)(2-((3-(p-tolyl)isoxazol-5-yl)methoxy)phenyl)methanone* (5b) :**

White solid; Yield = 75%; M.p.: 178–180 °C.

IR (KBr, νmax, cm^{-1}): 3115 (C4–H), 3112 (Ar–H), 2925, 2871 (C–H_{aliph}), 1661 (C=O), 1600 (C=N).

^{1}H NMR (300 MHz, $CDCl_3$) (δ in ppm): 2.31 (s, 3H, CH_3), 2.41 (s, 3H, CH_3), 6.43 (dd, 2H, J = 3 Hz, J = 0.9 Hz, CH_2), 6.41 (d, 1H, J = 10 Hz, CH), 6.39 (d, 1H, J = 10 Hz, Ar–H), 6.99 (d, 2H, J = 8 Hz, Ar–H), 7.09–7.22 (m, 7H, Ar–H), 7.24–7.37 (m, 4H, Ar–H), 7.38–7.65 (m, 5H, Ar–H).

^{13}C NMR (75 MHz, $CDCl_3$) (δ in ppm): 21.33 (CH_3), 21.44 (CH_3), 61.90 (O–CH_2), 101.22 (CH), 112.14, 121.93, 121.98, 124.83, 125.65, 126.72, 126.82, 127.67, 128.23, 128.39, 128.40, 128.43, 128.82, 129.19, 129.51, 130.05, 130.83, 134.28, 139.74, 140.13, 156.59, 162.08, 162.43, 163.01, 167.07, 183.51 (C=O).

HRMS (ESI, m/z): calculated mass for $[C_{34}H_{26}N_2O_4+H]^+$ = 527.19708; found = 527.19299; calculated for $[C_{34}H_{26}N_2O_4 + Na]^+$ = 549.17903; found = 549.17456.

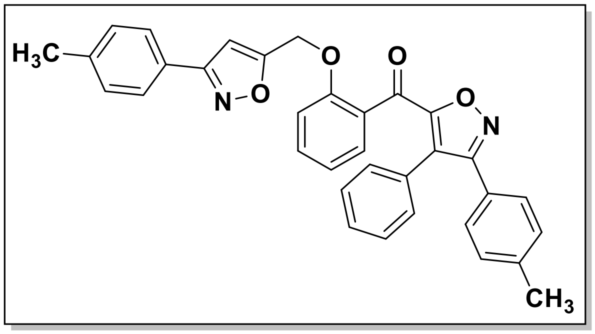

***(2-((3-(4-chlorophenyl)isoxazol-5-yl)methoxy)phenyl)(4-phenyl-3-(p-tolyl)isoxazol-5-yl)methanone* (5c) :**

White solid; Yield = 68%; **M.p.**: 220–222 °C.

IR (KBr, νmax, cm⁻¹): 3129 (C4–H), 3039 (Ar–H), 2914, 2847 ($C–H_{aliph}$), 1664 (C=O), 1594 (C=N).

^{1}H NMR (300 MHz) (δ in ppm): 2.32 (s, 3H, CH_3), 5.11 (s, 2H, $O–CH_2$), 6.40 (d, 1H, CH), 6.39 (d, 1H, *J* = 0.9 Hz, Ar–H), 6.93 (d, 2H, *J* = 8.4 Hz, Ar–H), 6.98 (d, 2H, *J* = 8.1 Hz, Ar–H), 7.11–7.19 (m, 5H, Ar–H), 7.23–7.36 (m, 5H, Ar–H), 7.49–7.65 (m, 4H, Ar–H).

^{13}C NMR (75 MHz) (δ in ppm): 21.32 (CH_3), 61.77 ($O–CH_2$), 101.27 (CH), 112.13, 121.82, 122.10, 124.76, 126.95, 127.68, 128.03, 128.26, 128.30, 128.31, 128.33, 128.44, 129.05, 129.20, 129.99, 130.90, 134.34, 136.01, 139.95, 156.60, 161.50, 161.96, 163.14, 167.55, 183.44 (C=O).

HRMS (ESI, m/z): calculated mass for $[C_{33}H_{24}ClN_2O_4 + H]^+$ = 548.01500; found = 547.12909, calculated mass for $[C_{33}H_{24}ClN_2O_4 + Na]^+$ = 569.12383; found = 569.11047.

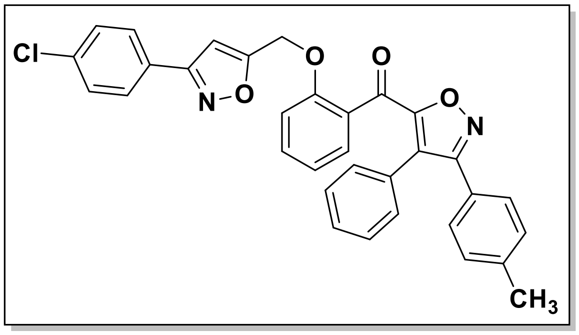

***(4-phenyl-3-(p-tolyl)isoxazol-5-yl)(2-((3-(4-(trifluoromethyl)phenyl)isoxazol-5-yl)methoxy)phenyl)methanone* (5d):**

White solid; Yield = 85%; **M.p.:** 228–230 °C.

IR (KBr, νmax, cm^{-1}): 3141 (C4–H), 3048 (Ar–H), 2930, 2873 (C–H_{aliph}), 1661 (C=O), 1599 (C=N).

^{1}H NMR (300 MHz, $CDCl_3$) (δ in ppm): 2.29 (s, 3H, CH_3), 5.14 (s, 2H, O–CH_2), 6.47 (s, 1H, CH), 6.48 (d, 1H, J = 4.2 Hz, Ar–H), 6.96 (dd, 2H, J = 3.6, 8.7 Hz, Ar–H), 7.12–7.19 (m, 5H, Ar–H), 7.24–7.35 (m, 3H, Ar–H), 7.30 (dd, 4H, J = 8.1 Hz, Ar–H), 7.51–7.66 (m, 4H, Ar–H).

^{13}C NMR (75 MHz, $CDCl_3$) (δ in ppm): 21.19 (CH_3), 61.73 (O–CH_2), 101.45 (CH), 112.13, 121.84, 122.18, 124.74, 125.70, 125.75, 127.08, 127.67, 128.26, 128.34, 128.48, 129.21, 129.97, 130.95, 131.86, 134.39, 139.98, 156.58, 161.33, 161.50, 161.93, 163.16, 167.88, 183.40 (C=O).

HRMS (ESI, m/z): calculated mass for $[C_{34}H_{24}F_3N_2O_4+H]^+$ = 581.57121; found = 581.15222, calculated mass for $[C_{34}H_{24}F_3N_2O_4+Na]^+$ = 603.09912; found = 603.13306.

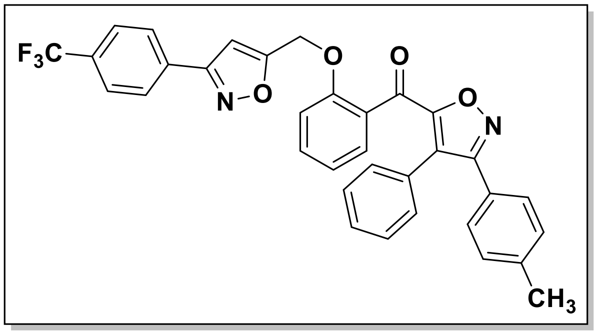

(3-(4-chlorophenyl)-4-phenylisoxazol-5-yl)(2-((3-phenylisoxazol-5-yl)methoxy)phenyl)methanone **(5e) :**

White solid; Yield = 70%; **M.p.**: 174–176 °C.

IR (KBr, νmax, cm^{-1}): 3135 (C4–H), 3054 (Ar–H), 2918, 2870 (C–H_{aliph}), 1654 (C=O), 1593 (C=N).

^{1}H NMR (300 MHz, $CDCl_3$) (δ in ppm): 5.23 (d, 2H, J = 12.9 Hz, O–CH_2), 6.96 (s, 1H, CH), 7.08–7.34 (m, 11H, Ar–H), 7.44–7.54 (m, 2H, Ar–H), 7.56–7.67 (m, 2H, Ar–H), 7.71–7.74 (m, 2H, Ar–H), 7.88 (dd, 1H, J = 8.1, 30.7 Hz, Ar–H).

^{13}C NMR (75 MHz, $CDCl_3$) (δ in ppm): 61.31 (O–CH_2), 102.32 (CH), 113.29, 121.35, 122.11, 126.60, 126.95, 126.99, 127.72, 127.77, 127.82, 128.53, 128.72, 128.80, 129.04, 129.07, 129.11, 129.51, 130.25, 130.40, 130.60, 130.76, 135.26, 135.33, 156.93, 161.04, 162.20, 163.40, 167.83, 183.82 (C=O).

HRMS (ESI, m/z): calculated mass for $[C_{32}H_{22}ClN_2O_4 + H]^+$ = 533.12572; found = 533.11389, calculated mass for $[C_{32}H_{22}ClN_2O_4 + Na]^+$ = 555.10766; found = 555.09521.

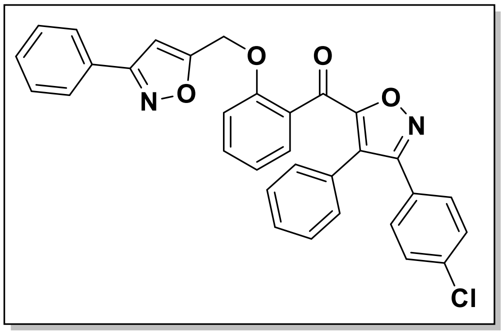

(3-(4-chlorophenyl)-4-phenylisoxazol-5-yl)(2-((3-(p-tolyl)isoxazol-5-yl)methoxy)phenyl)methanone **(5f):**

White solid; Yield = 73%; M.p.: 196–198 °C.

IR (KBr, νmax, cm^{-1}): 3112 (C4–H), 3052 (Ar–H), 2920, 2870 (C–H_{aliph}), 1664 (C=O), 1600 (C=N).

^{1}H NMR (300 MHz, $CDCl_3$) (δ in ppm): 2.42 (s, 3H, CH_3), 5.08 (d, 2H, O–CH_2), 6.39 (s, 1H, CH), 6.93 (dd, 1H, J = 0.6, 8.4 Hz, Ar–H), 7.09–7.22 (m, 9H, Ar–H), 7.24–7.32 (m, 3H, Ar–H), 7.49–7.56 (m, 3H, Ar–H), 7.65 (dd, 1H, J = 1.8, 7.8 Hz, Ar–H).

^{13}C NMR (75 MHz, $CDCl_3$) (δ in ppm): 21.45 (CH_3), 61.66 (O–CH_2), 101.41 (CH), 112.08, 121.52, 122.05, 125.49, 126.17, 126.57, 127.45, 127.83, 128.42, 128.61, 128.72, 129.57, 129.76, 129.98, 130.91, 134.54, 135.92, 140.39, 156.76, 161.01, 162.34, 163.48, 166.82, 183.35 (C=O).

HRMS (m/z): calculated mass for $[C_{33}H_{24}ClN_2O_4+H]^+$ = 547.14137; found = 547.12903, calculated mass for $[C_{33}H_{24}ClN_2O_4+Na]^+$ = 569.12331; found = 569.11035.

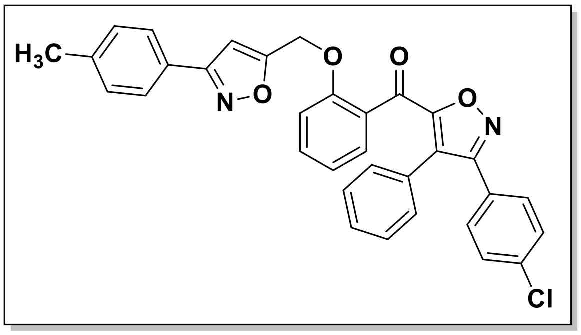

(3-(4-chlorophenyl)-4-phenylisoxazol-5-yl)(2-((3-(4-chlorophenyl)isoxazol-5-yl)methoxy)phenyl)methanone **(5g) :**

White solid; Yield = 72%; **M.p.: 164–166 °C.**

IR (KBr, νmax, cm^{-1}): 3120 (C4–H), 3052 (Ar–H), 2920, 2848 (aliphatic C–H), 1667 (C=O), 1600 (C=N).

^{1}H NMR (300 MHz, $CDCl_3$) (δ in ppm): 5.10 (s, 2H, O–CH_2), 6.41 (s, 1H, CH), 6.93 (dd, 1H, *J* = 0.9, 8.4 Hz, Ar–H), 7.11–7.17 (m, 5H, Ar–H), 7.19–7.33 (m, 4H, Ar–H), 7.36–7.39 (m, 2H, Ar–H), 7.51–7.58 (m, 3H, Ar–H), 7.65 (dd, 1H, *J* = 1.8, 7.5 Hz, Ar–H).

^{13}C NMR (75 MHz, $CDCl_3$) (δ in ppm): 61.62 (O–CH_2), 101.37 (CH), 112.11, 122.15, 126.83, 127.46, 127.81, 127.92, 128.47, 128.78, 129.17, 129.71, 129.94, 130.94, 134.54, 136.09, 136.29, 156.66, 160.96, 161.42, 163.46, 167.38, 183.26 (C=O).

HRMS (m/z): calculated mass for $[C_{32}H_{21}Cl_2N_2O_4+H]^+$ = 567.08784; found = 567.07416, calculated mass for $[C_{32}H_{21}Cl_2N_2O_4+Na]^+$ = 589.0686; found = 589.05542.

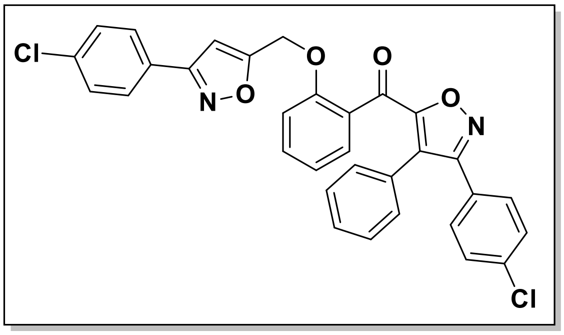

(3-(4-chlorophenyl)-4-phenylisoxazol-5-yl)(2-((3-(p-tolyl)isoxazol-5-yl)methoxy)phenyl)methanone **(5h):**

White solid; Yield = 82%; M.p.: 242–244 °C.

IR (KBr, νmax, cm^{-1}): 3111 (C4–H), 3058 (Ar–H), 2914, 2851 (aliphatic C–H), 1666 (C=O), 1596 (C=N).

^{1}H NMR (300 MHz, $CDCl_3$, δ in ppm): 5.23 (d, 2H, J = 12.9 Hz, O–CH_2), 6.96 (s, 1H, CH), 7.08–7.34 (m, 12H, Ar–H), 7.44–7.52 (m, 2H, Ar–H), 7.57–7.67 (m, 2H, Ar–H), 7.71–7.74 (m, 1H, Ar–H), 7.82 (d, 1H, J = 8.4 Hz, Ar–H), 7.93 (d, 1H, J = 8.1 Hz, Ar–H).

^{13}C NMR (75 MHz, $CDCl_3$, δ in ppm): 61.32 (O–CH_2), 102.75 (CH), 121.11, 121.36, 122.11, 126.51, 126.60, 126.95, 126.99, 127.72, 127.77, 127.82, 128.53, 128.72, 128.80, 129.05, 129.11, 129.51, 130.17, 130.25, 130.40, 130.60, 130.72, 130.77, 135.26, 156.81, 156.93, 161.05, 161.19, 163.59, 167.84, 183.82 (C=O).

HRMS (m/z): calculated mass for $[C_{33}H_{20}F_3N_2O_4 + H]^+$ = 601.11327; found = 601.10004, calculated mass for $[C_{33}H_{20}F_3N_2O_4 + Na]^+$ = 623.09504; found = 623.08142.

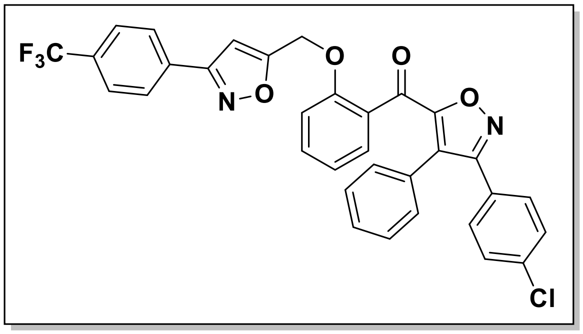

4.1 Copies of the (1H, 13C) NMR, IR and HRMS spectra of all new hybrid compounds (5a-h)

(4-phenyl-3-(p-tolyl)isoxazol-5-yl)(2-((3-phenylisoxazol-5-yl)methoxy)phenyl)methanone **(5a):**

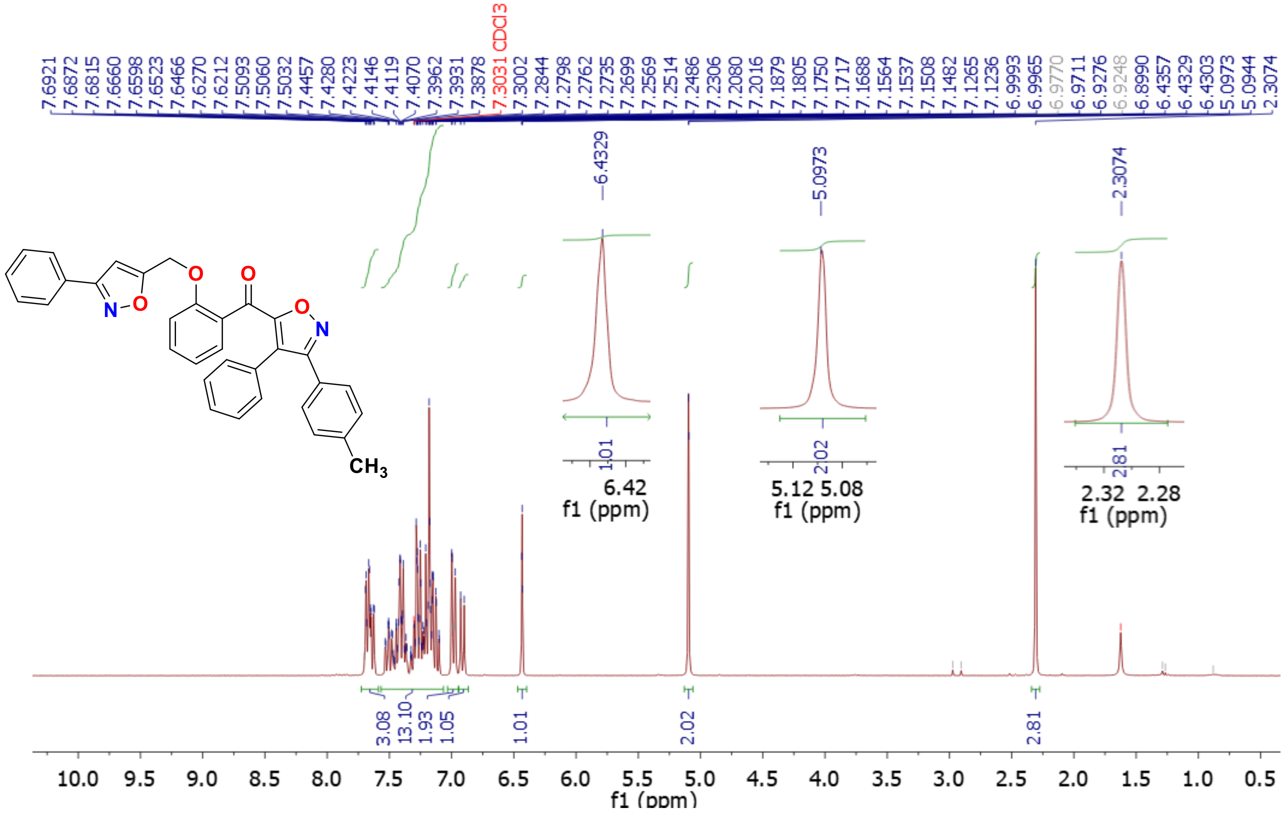

Figure S1. ^{1}H NMR spectrum (300 MHz, $CDCl_3$) of compound **5a**

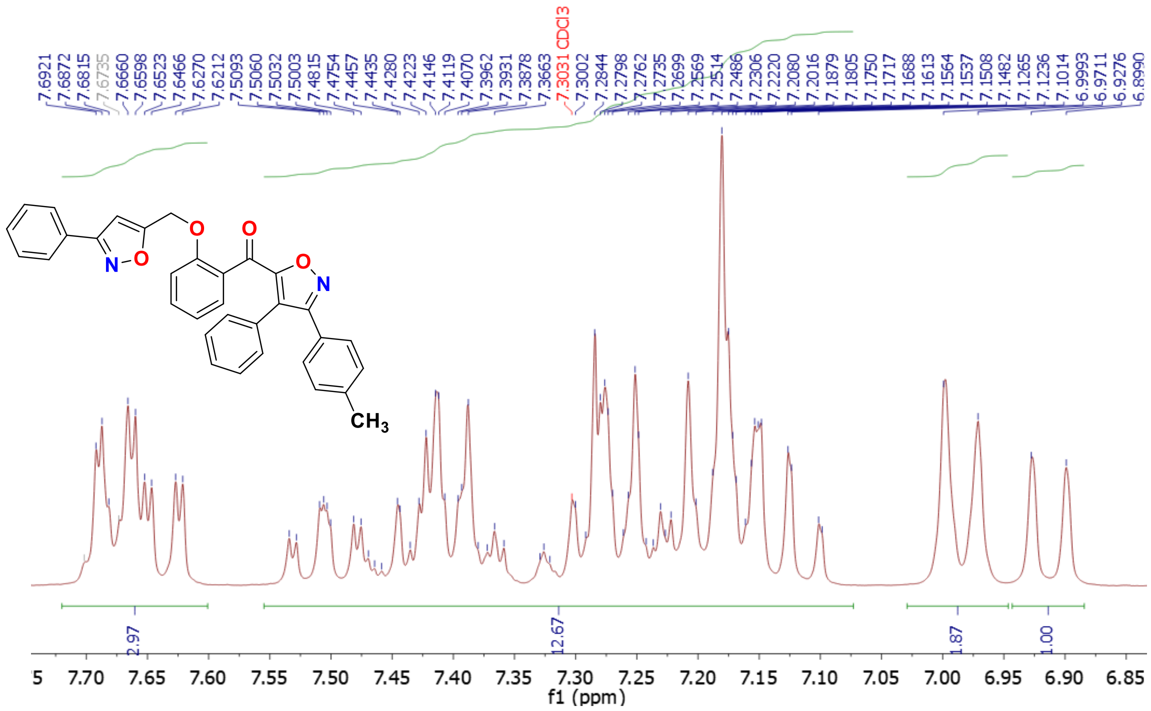

Figure S2. Aromatic enlarged region of ^{1}H NMR spectrum of compound **5a**

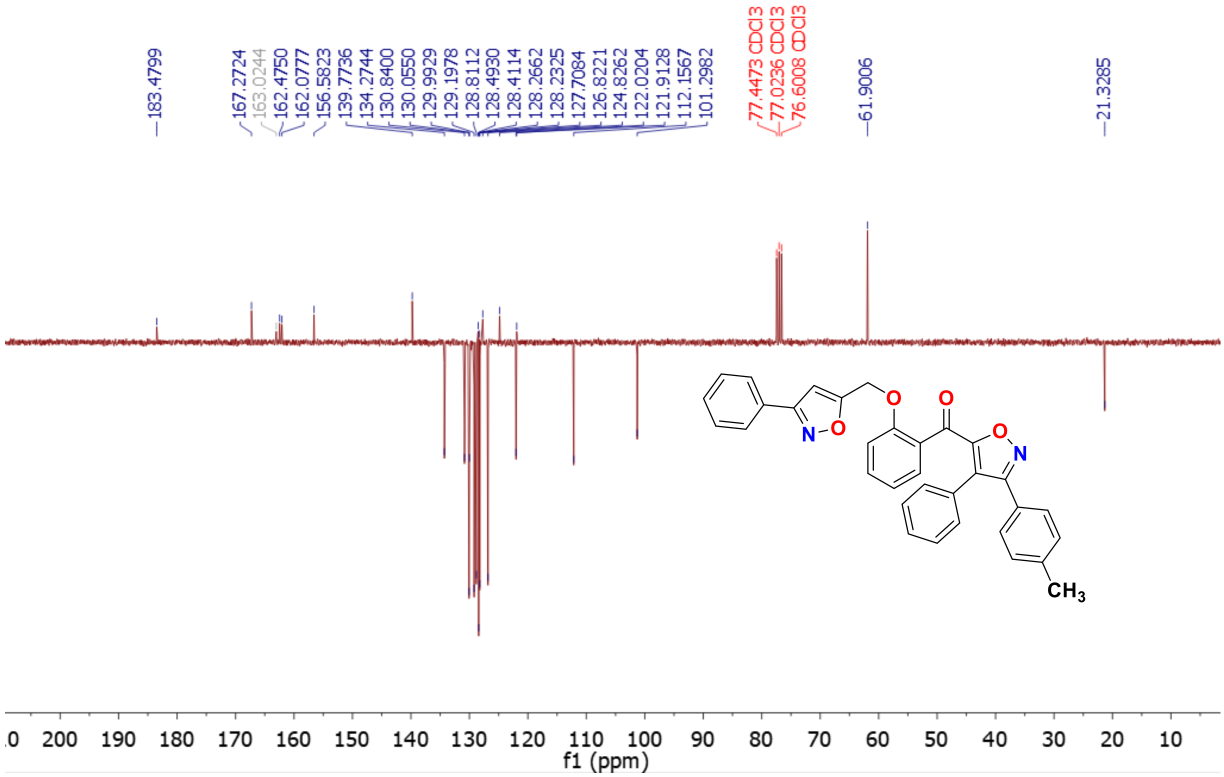

Figure S3. ^{13}C NMR spectrum (75 MHz, $CDCl_3$) of compound **5a**

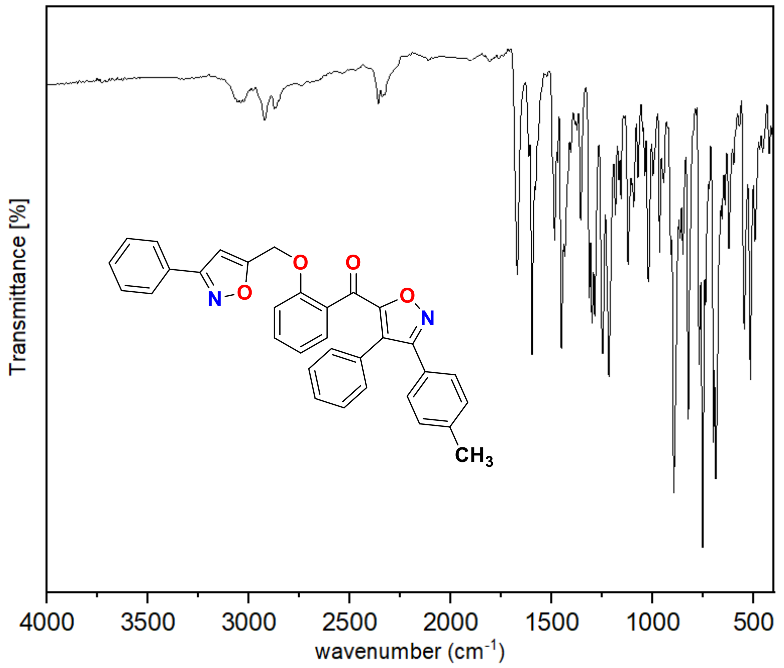

Figure S4. FT-IR spectrum of compound **5a**

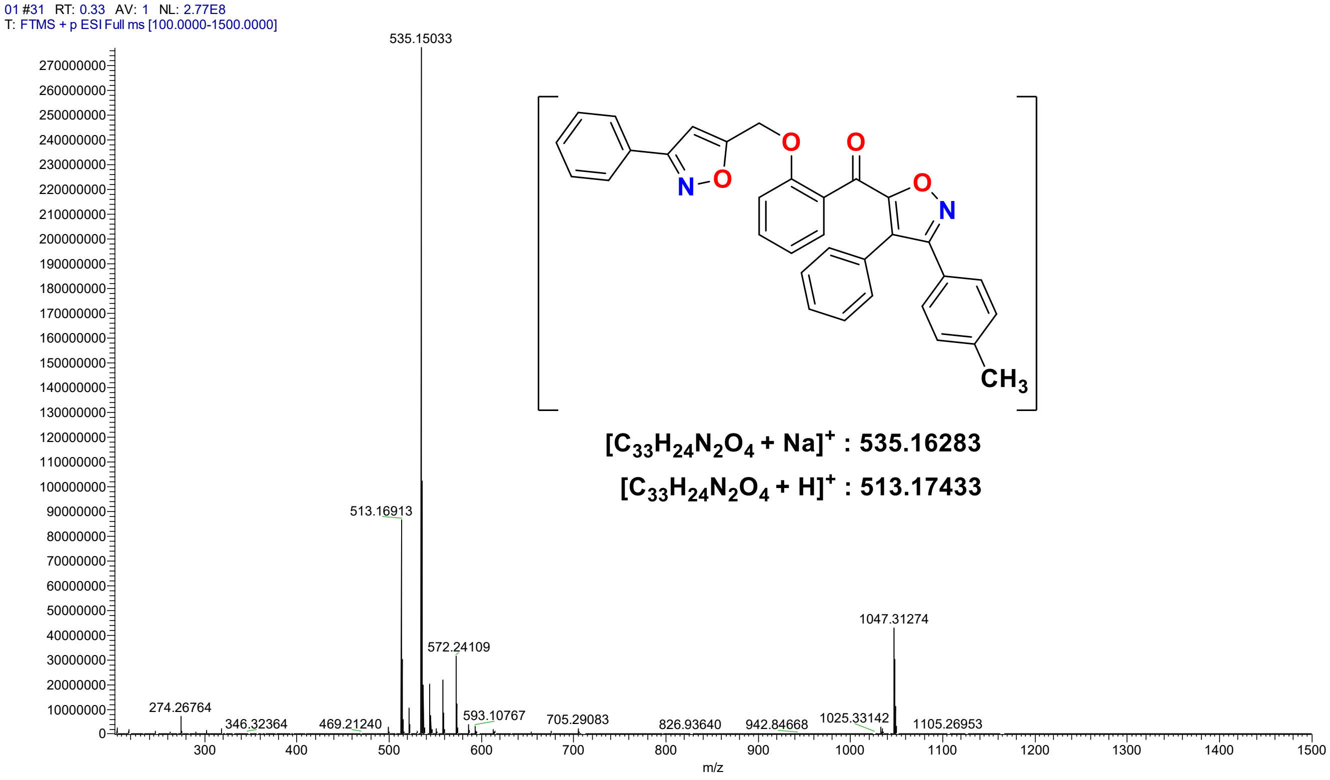

Figure S5. HRMS spectrum of compound **5a**

***(4-phenyl-3-(p-tolyl)isoxazol-5-yl)(2-((3-(p-tolyl)isoxazol-5-yl)methoxy)phenyl)methanone* ((b):**

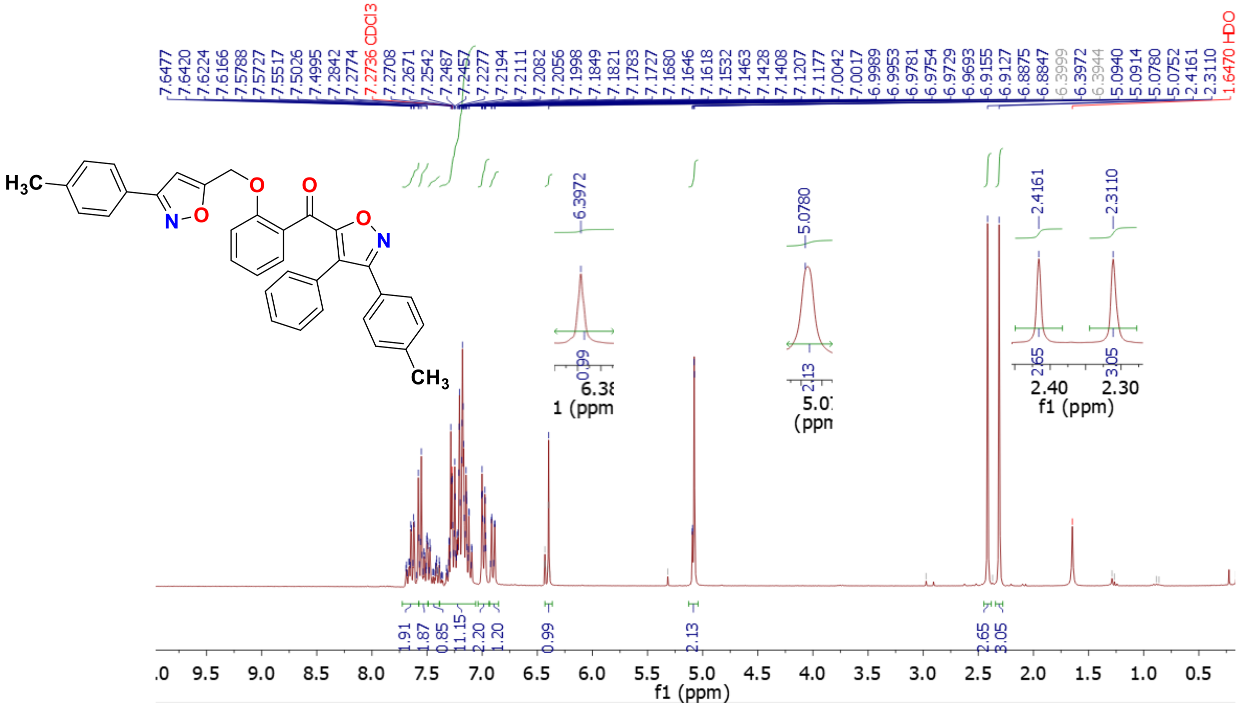

Figure S6. ^{1}H NMR spectrum (300 MHz, $CDCl_3$) of compound **5b**

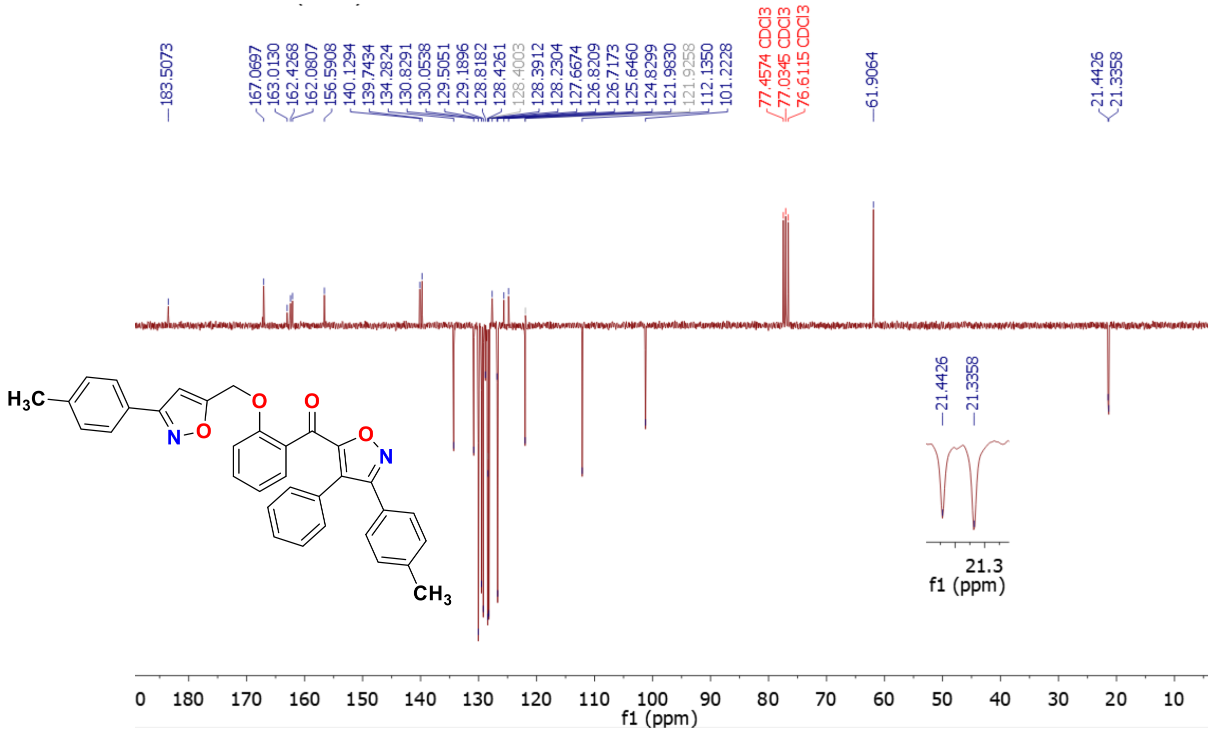

Figure S7. ^{13}C NMR spectrum (75 MHz, $CDCl_3$) of compound **5b**

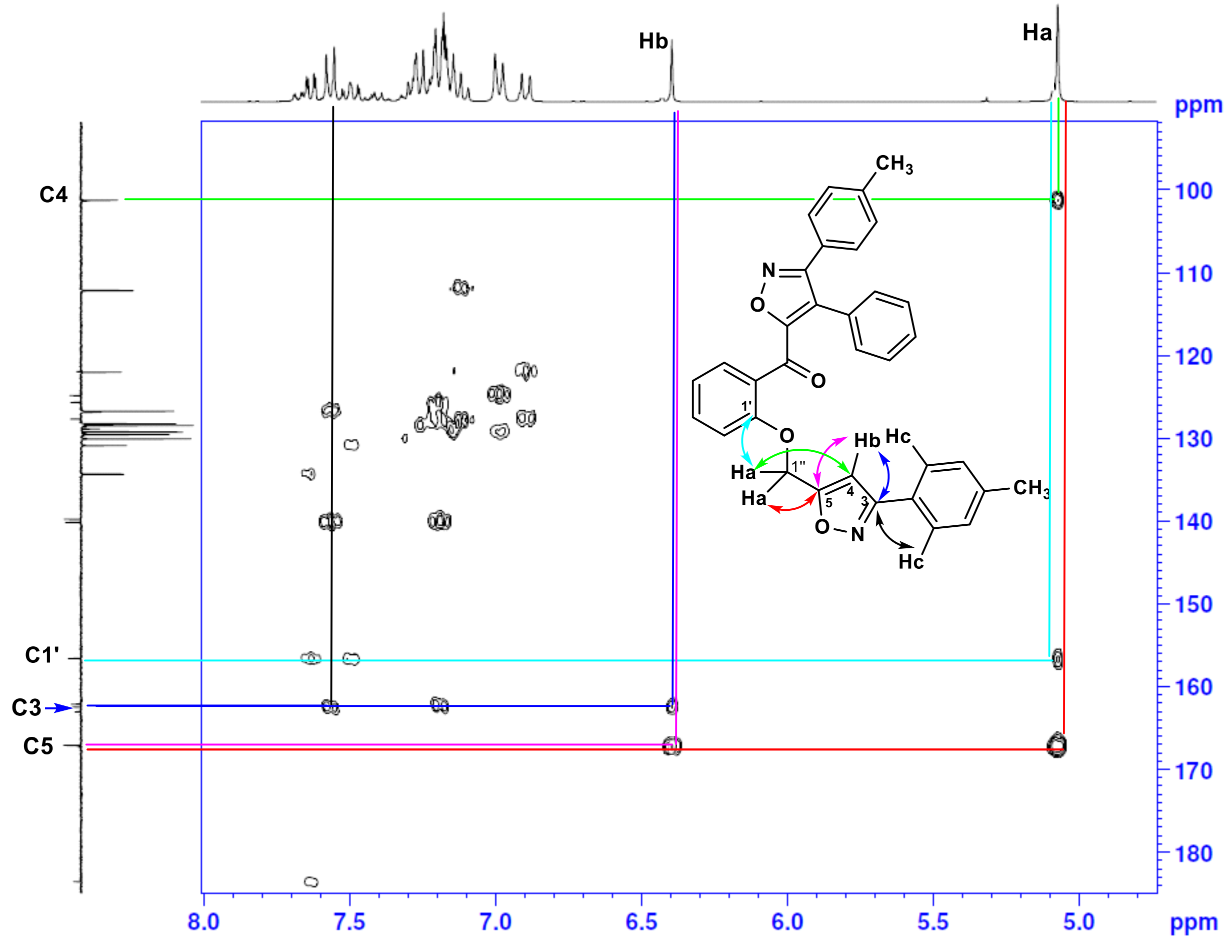

Figure S8. 2D-HMBC NMR spectrum of compound **5b** showing the long-range ^{1}H–^{13}C correlations

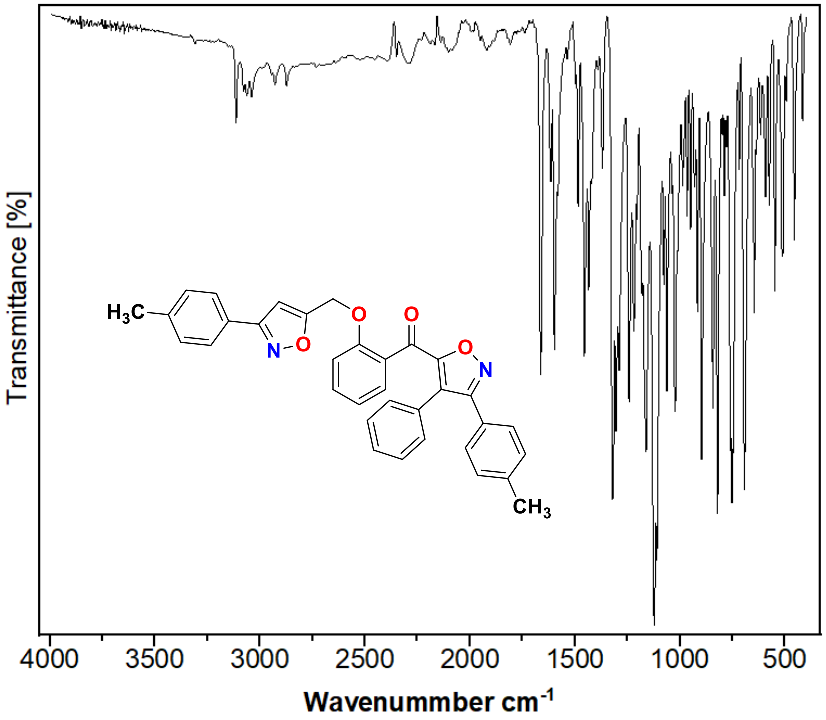

Figure S9. FT-IR spectrum of compound **5b**

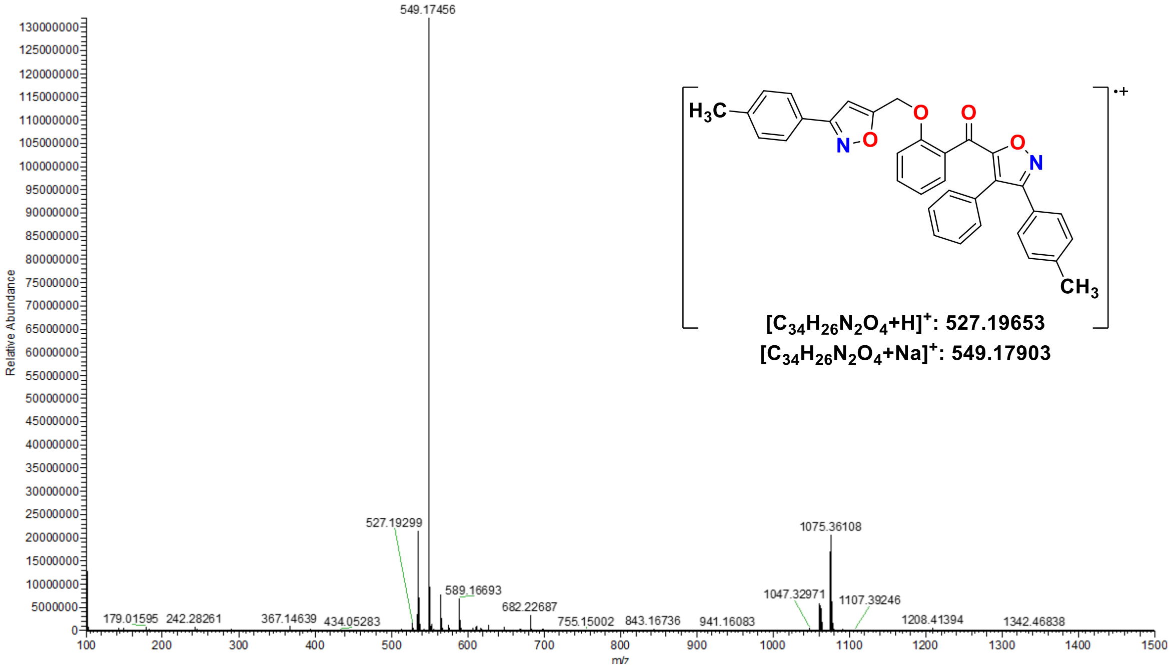

Figure S10. HRMS spectrum of compound **5b**

***(2-((3-(4-chlorophenyl)isoxazol-5-yl)methoxy)phenyl)(4-phenyl-3-(p-tolyl)isoxazol-5-yl)methanone* (5c) :**

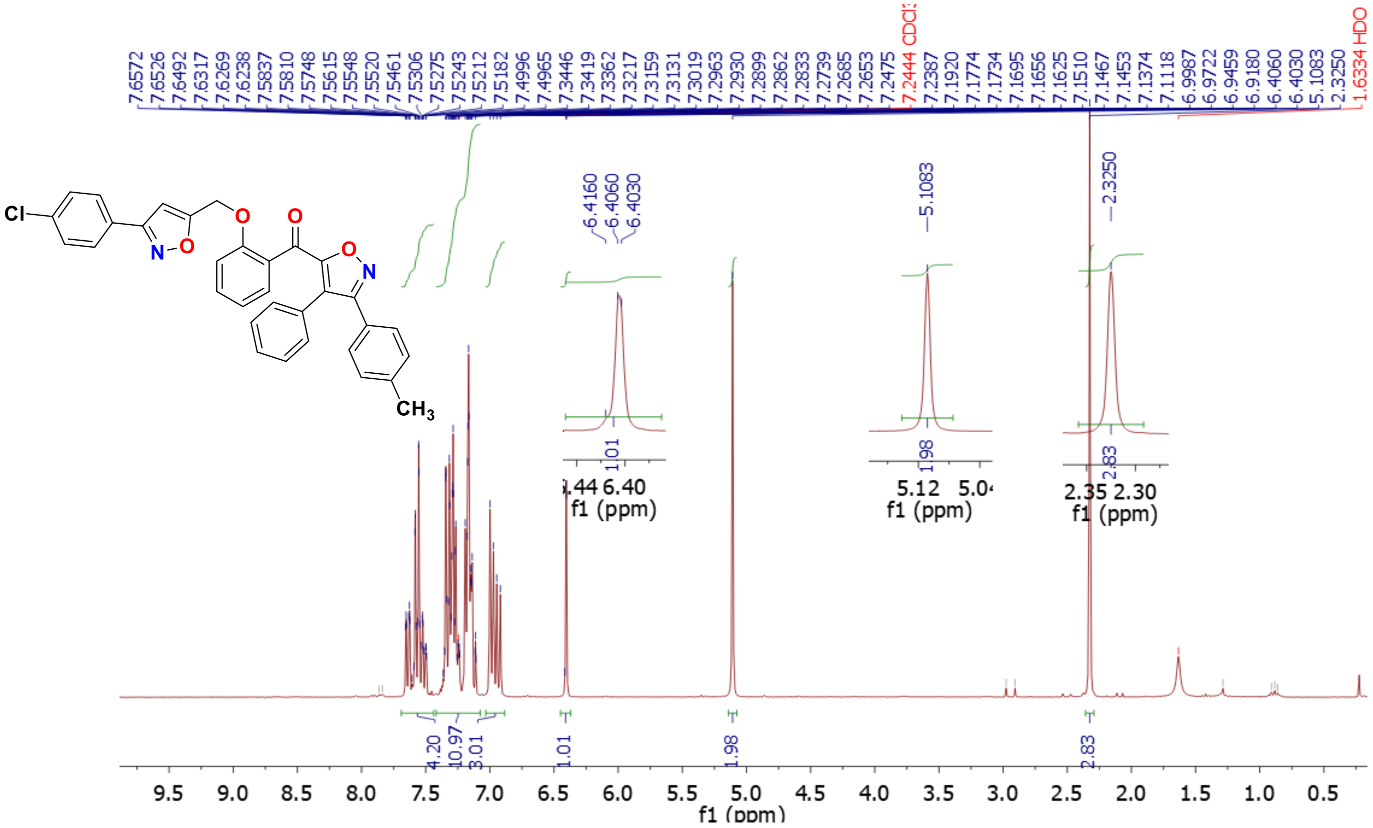

Figure S11. ^{1}H NMR spectrum (300 MHz, $CDCl_3$) of compound **5c**

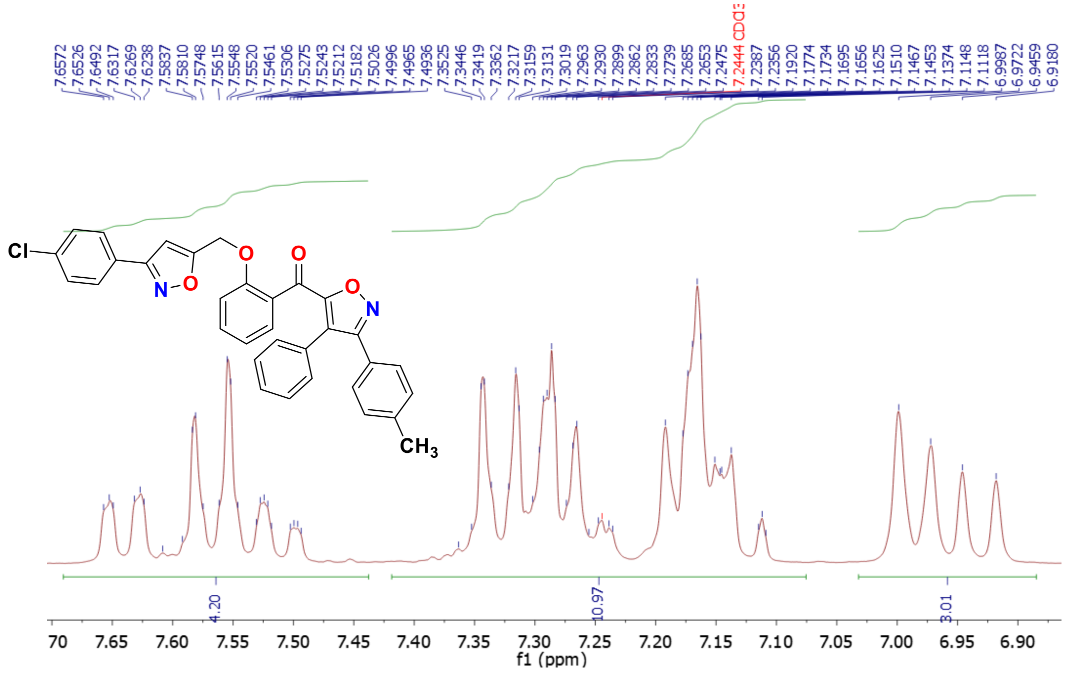

Figure S12. Aromatic enlarged region of ^{1}H NMR spectrum of compound **5c**

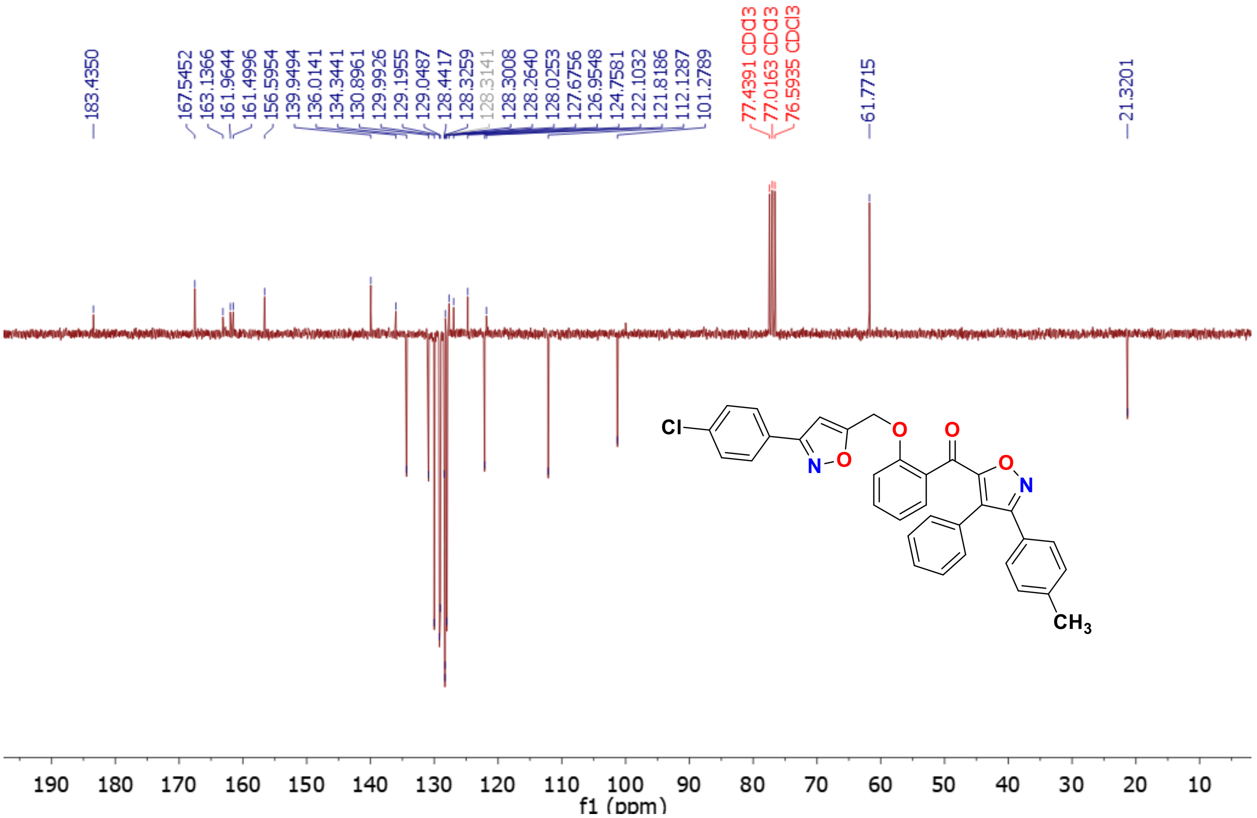

Figure S13. ^{13}C NMR spectrum (75 MHz, CDCl3) of compound **5c**

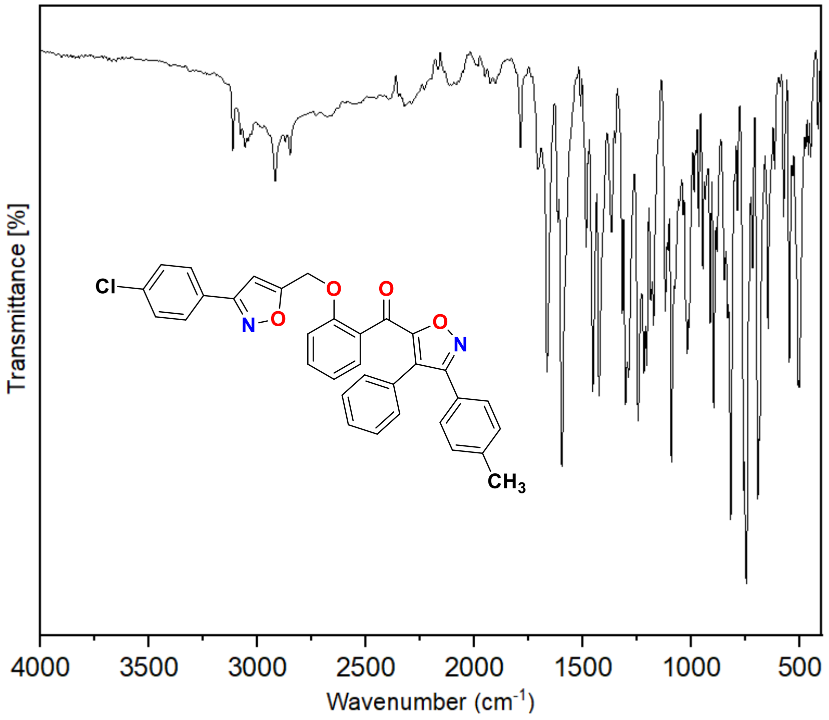

Figure S14. FT-IR spectrum of compound **5c**

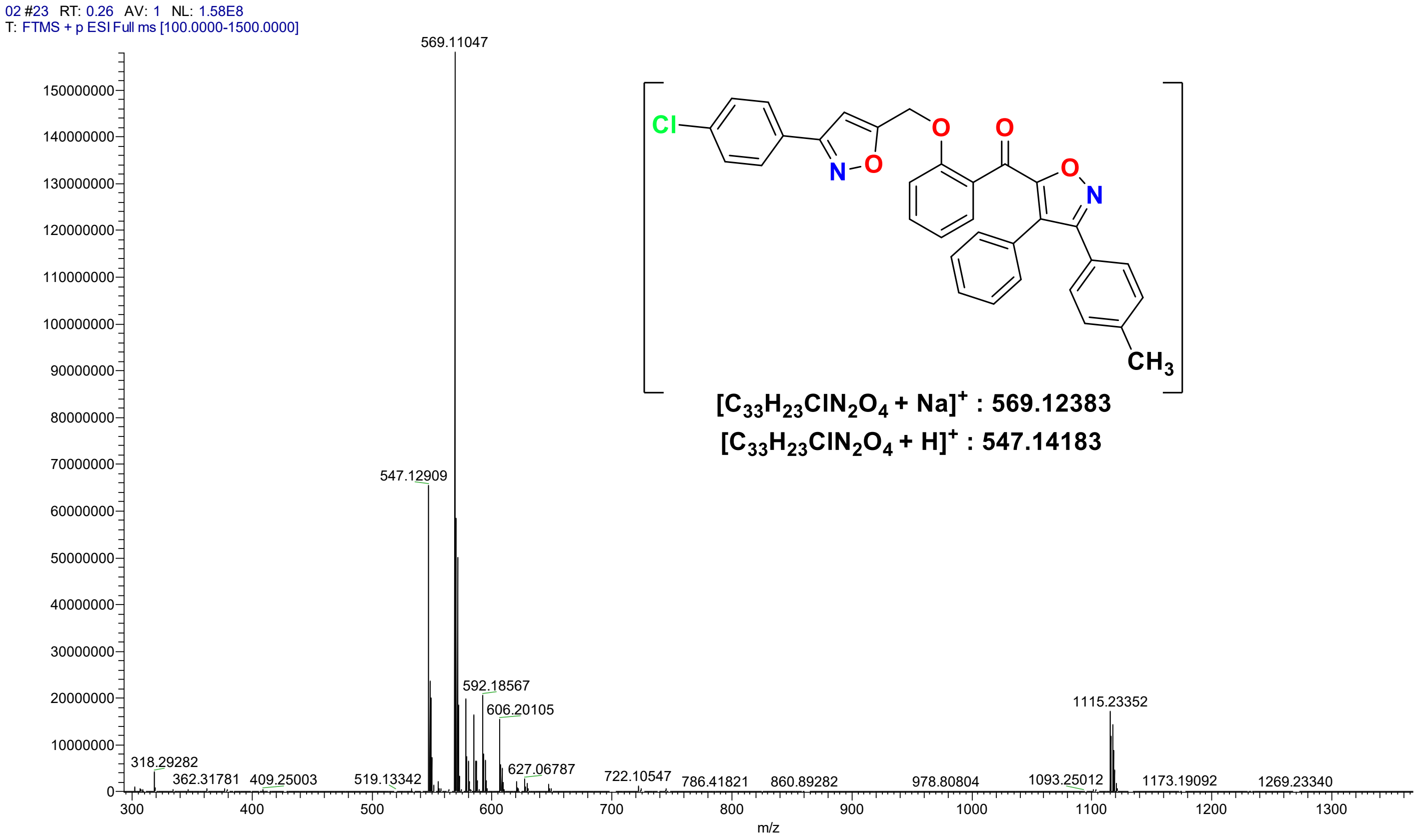

Figure S15. HRMS spectrum of compound **5c**

***(4-phenyl-3-(p-tolyl)isoxazol-5-yl)(2-((3-(4-(trifluoromethyl)phenyl)isoxazol-5-yl)methoxy)phenyl)methanone* (5d):**

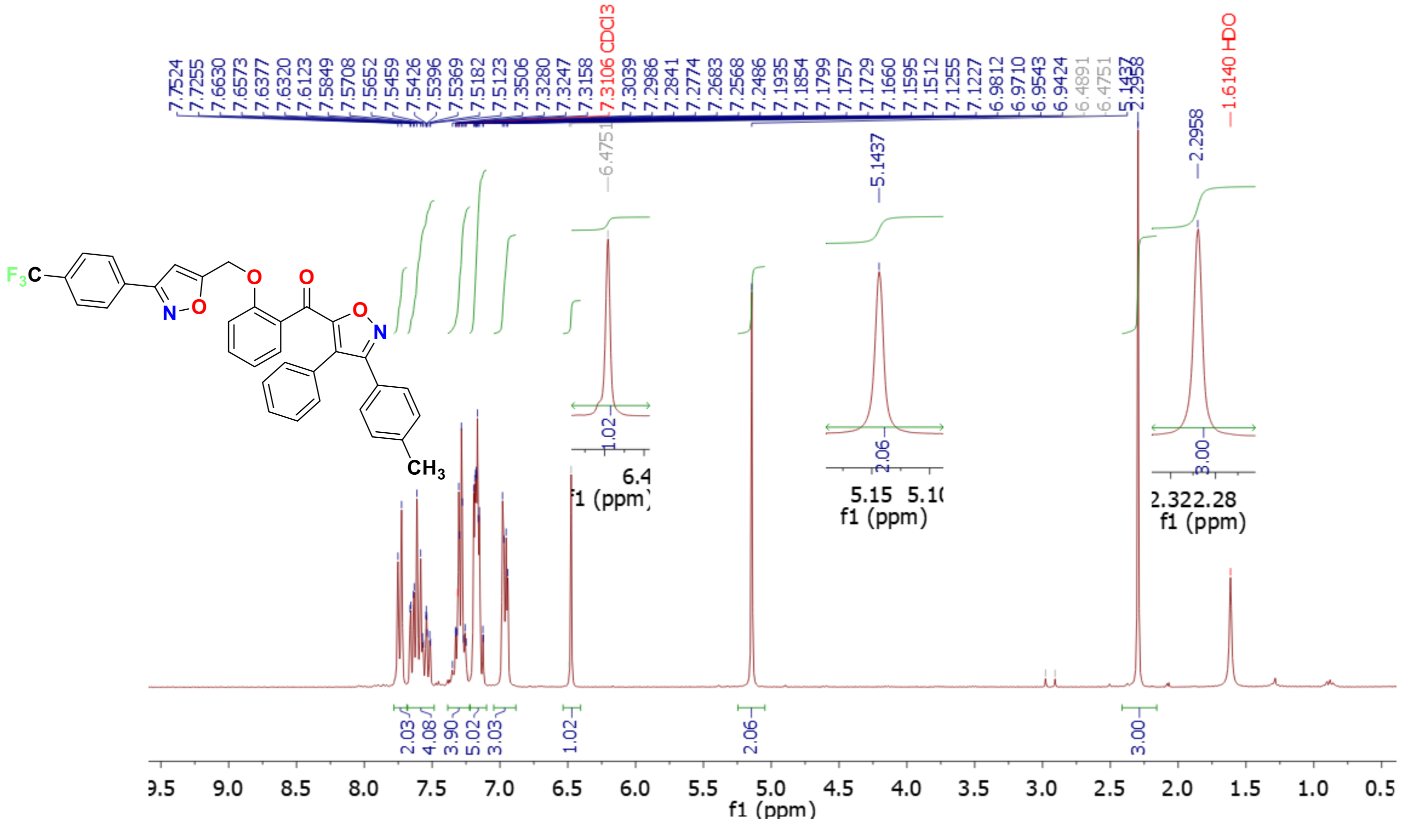

Figure S16. ^{1}H NMR spectrum (300 MHz, $CDCl_3$) of compound **5d**

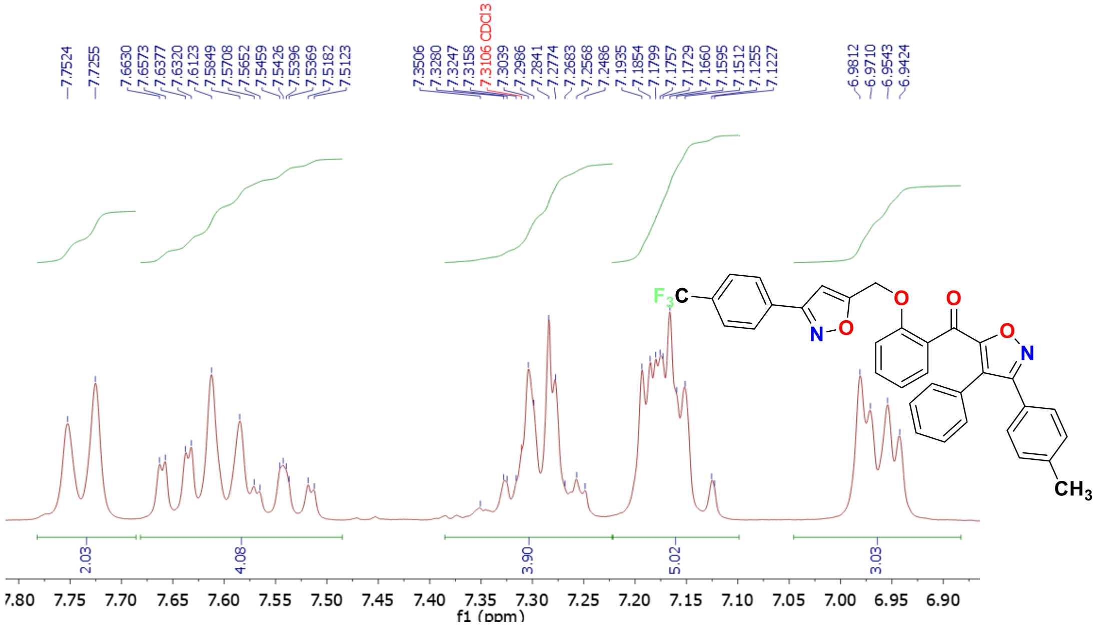

Figure S17. Aromatic enlarged region of ^{1}H NMR spectrum of compound **5d**

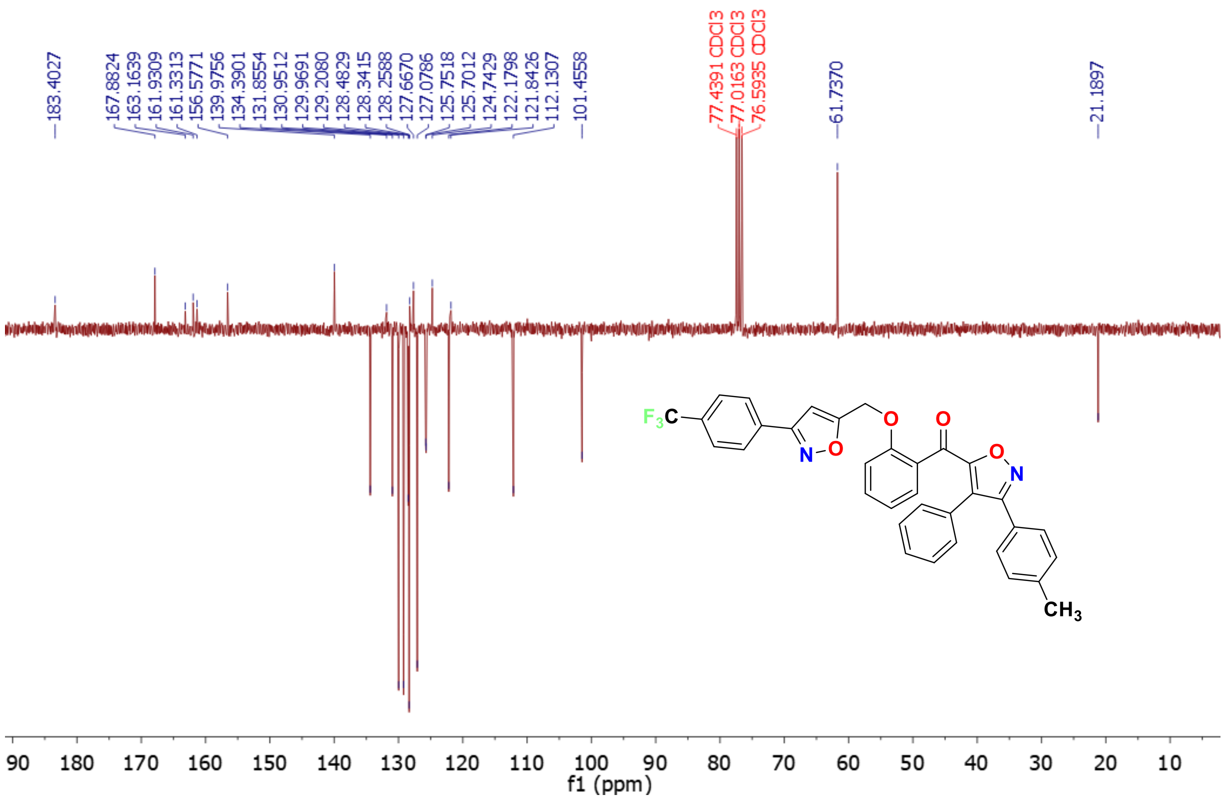

Figure S18. ^{13}C NMR spectrum (75 MHz, CDCl3) of compound **5d**

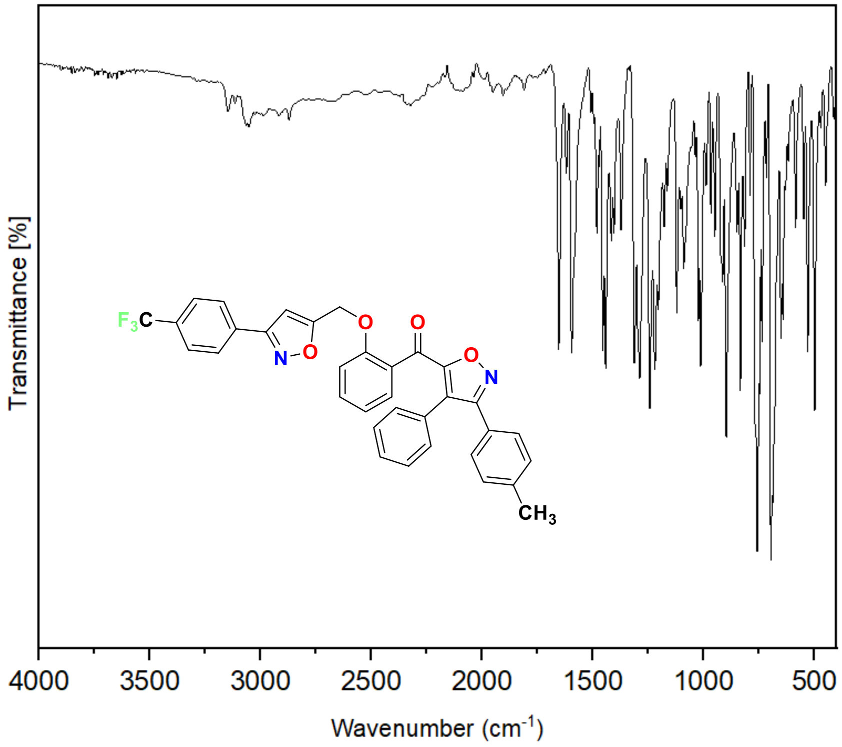

Figure S19. FT-IR spectrum of compound **5d**

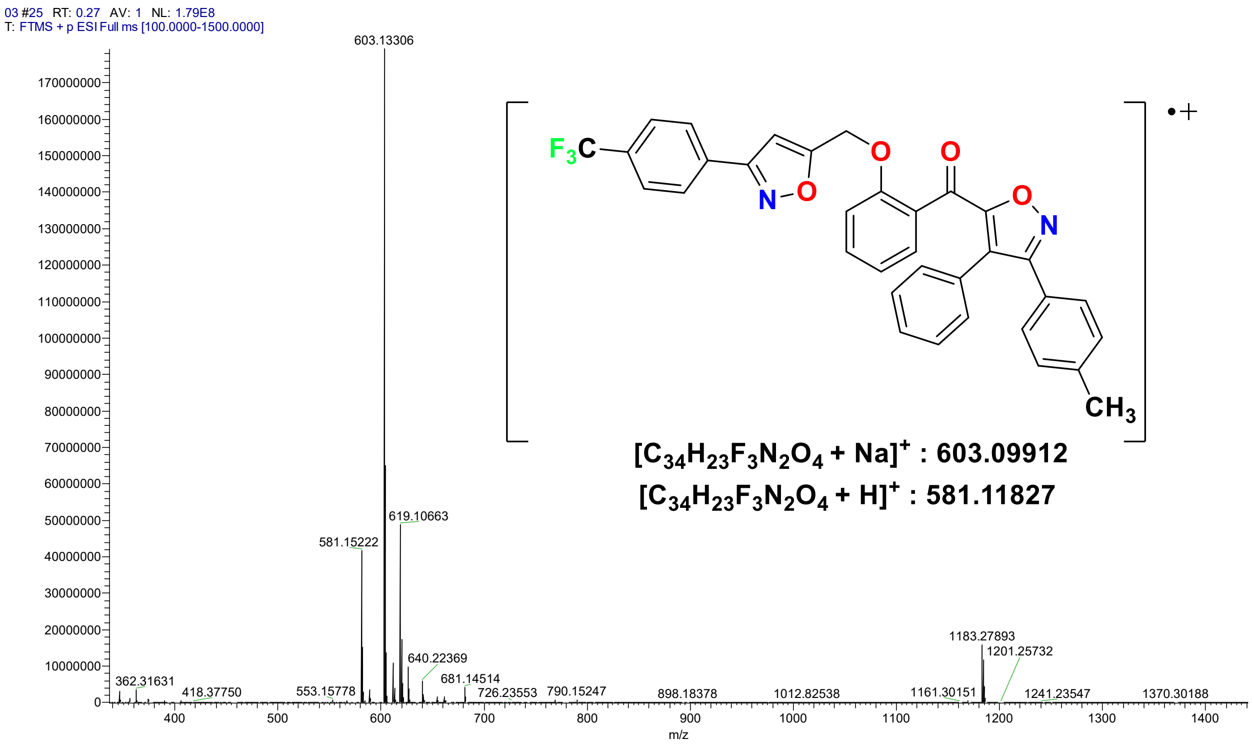

Figure S20. HRMS spectrum of compound **5d**

(3-(4-chlorophenyl)-4-phenylisoxazol-5-yl)(2-((3-phenylisoxazol-5-yl)methoxy)phenyl)methanone **(5e):**

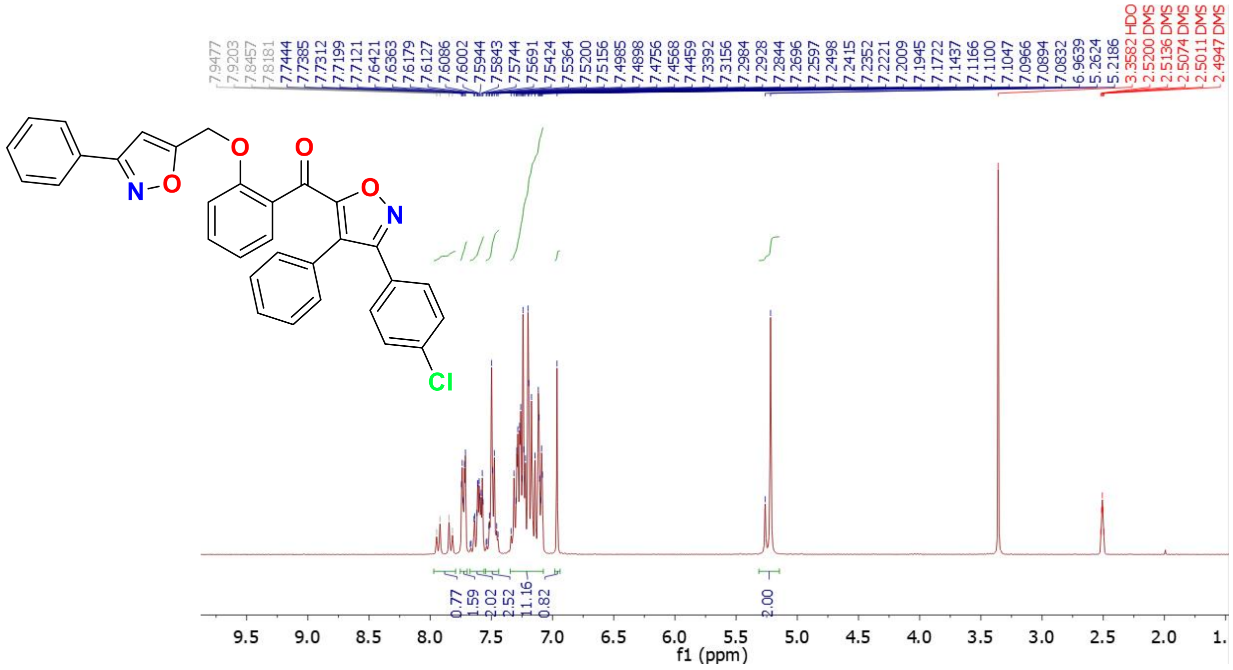

Figure S21. ^{1}H NMR spectrum (300 MHz, $CDCl_3$) of compound **5e**

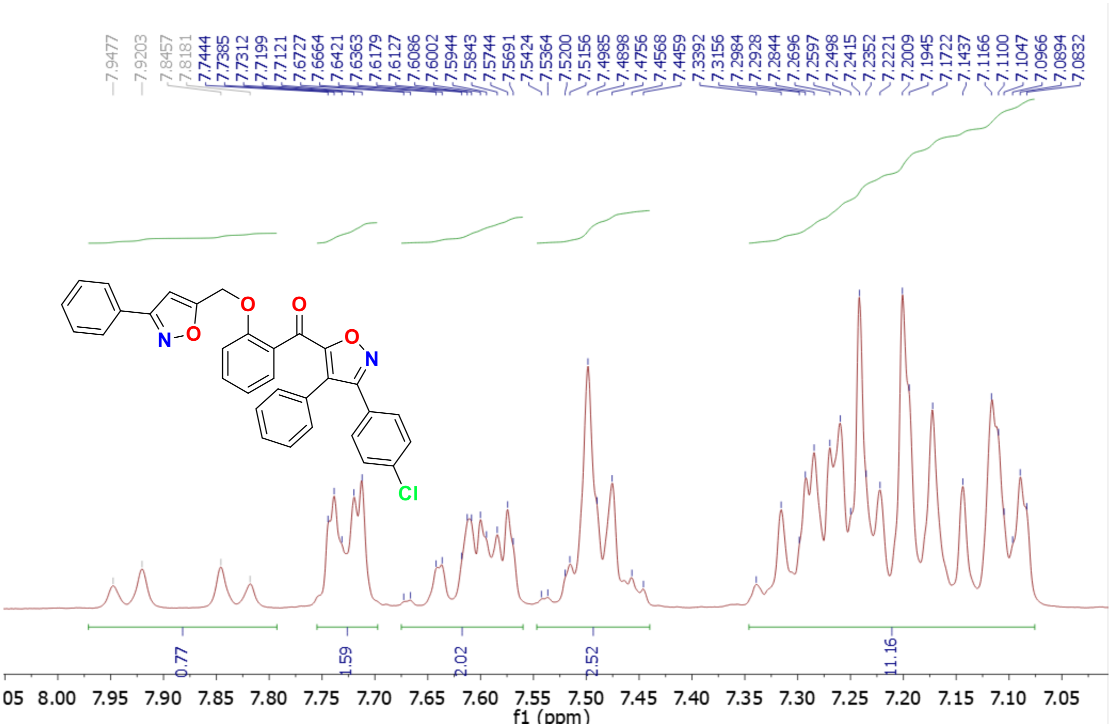

Figure S22. Aromatic enlarged region of ^{1}H NMR spectrum of compound **5e**

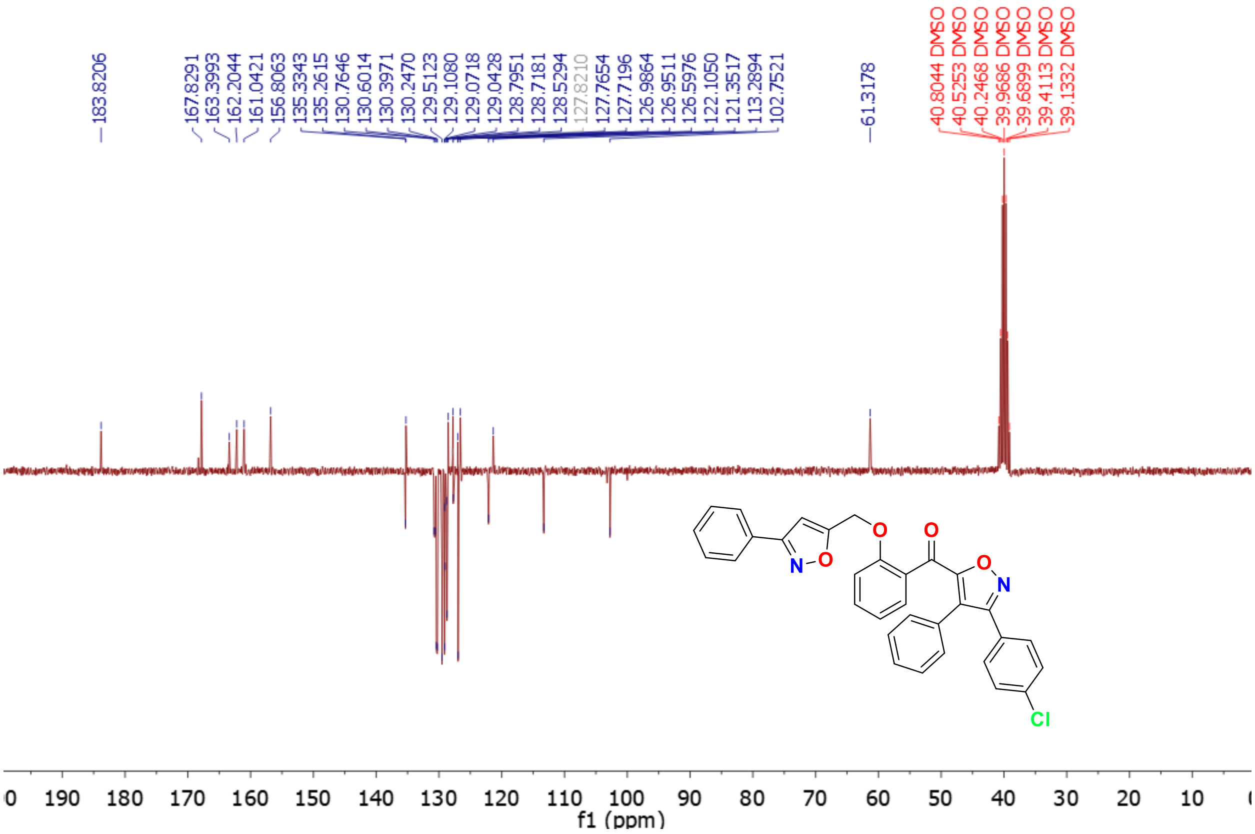

Figure S23. ^{13}C NMR spectrum (75 MHz, CDCl3) of compound **5e**

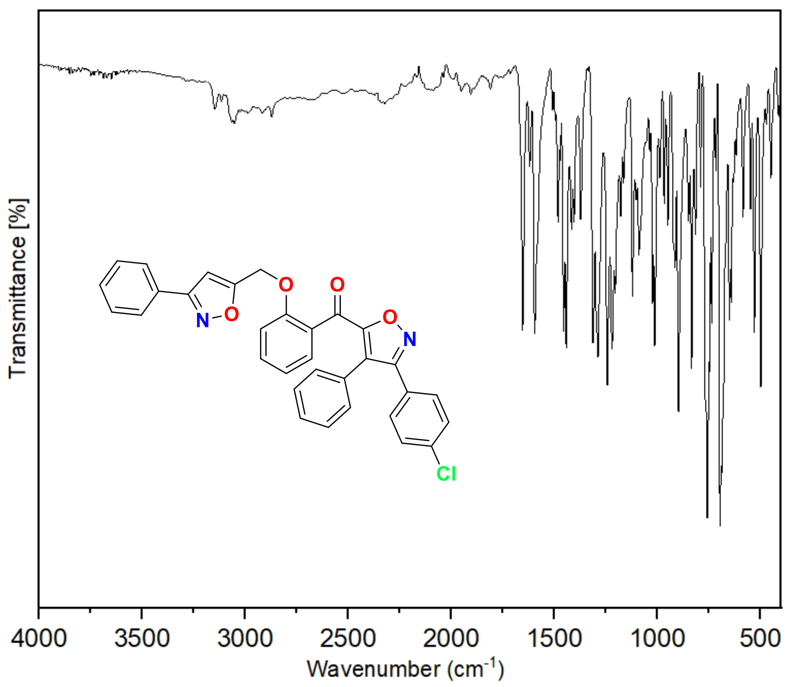

Figure S24. FT-IR spectrum of compound **5e**

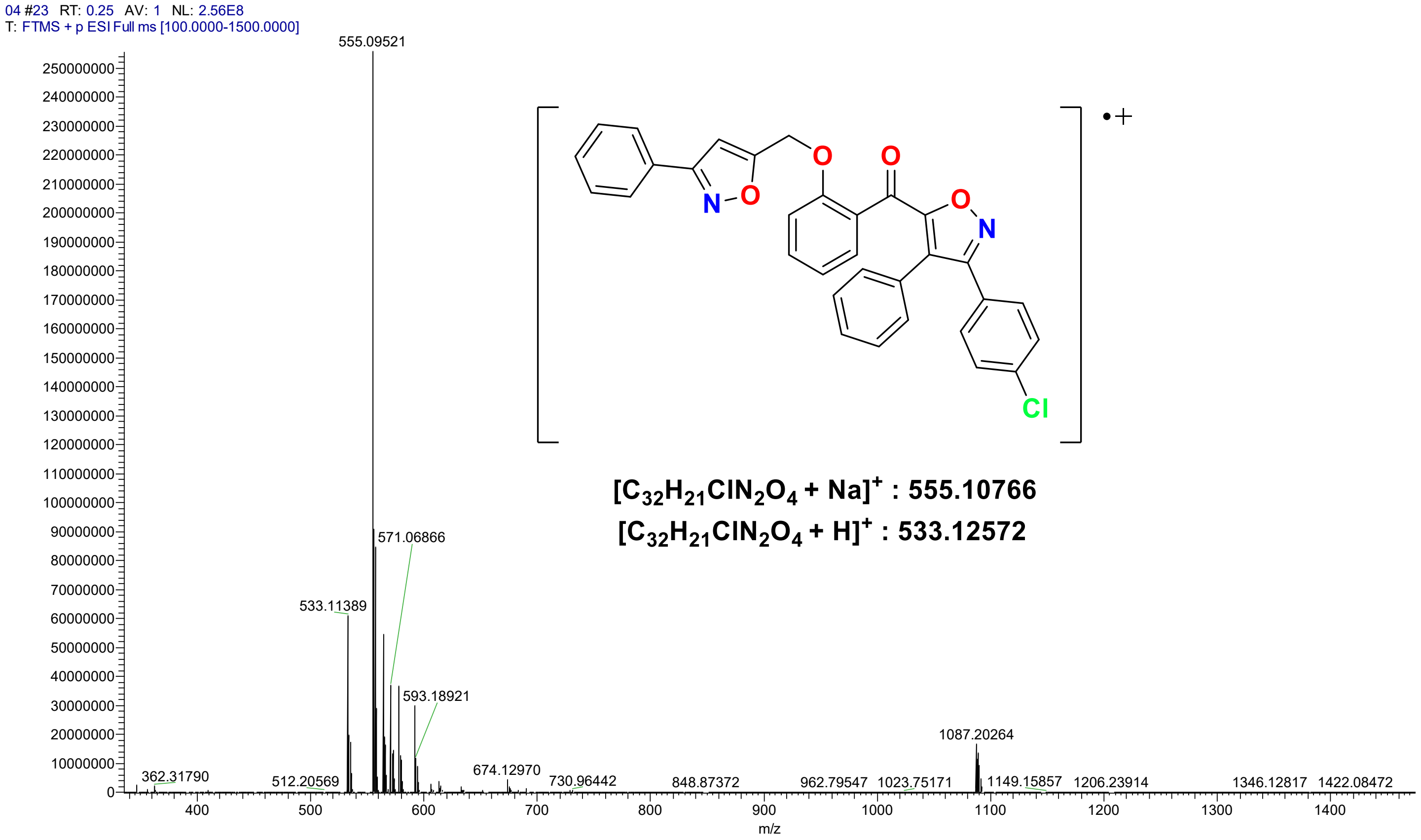

Figure S25. HRMS spectrum of compound **5e**

(3-(4-chlorophenyl)-4-phenylisoxazol-5-yl)(2-((3-(p-tolyl)isoxazol-5-yl)methoxy)phenyl)methanone (5f):

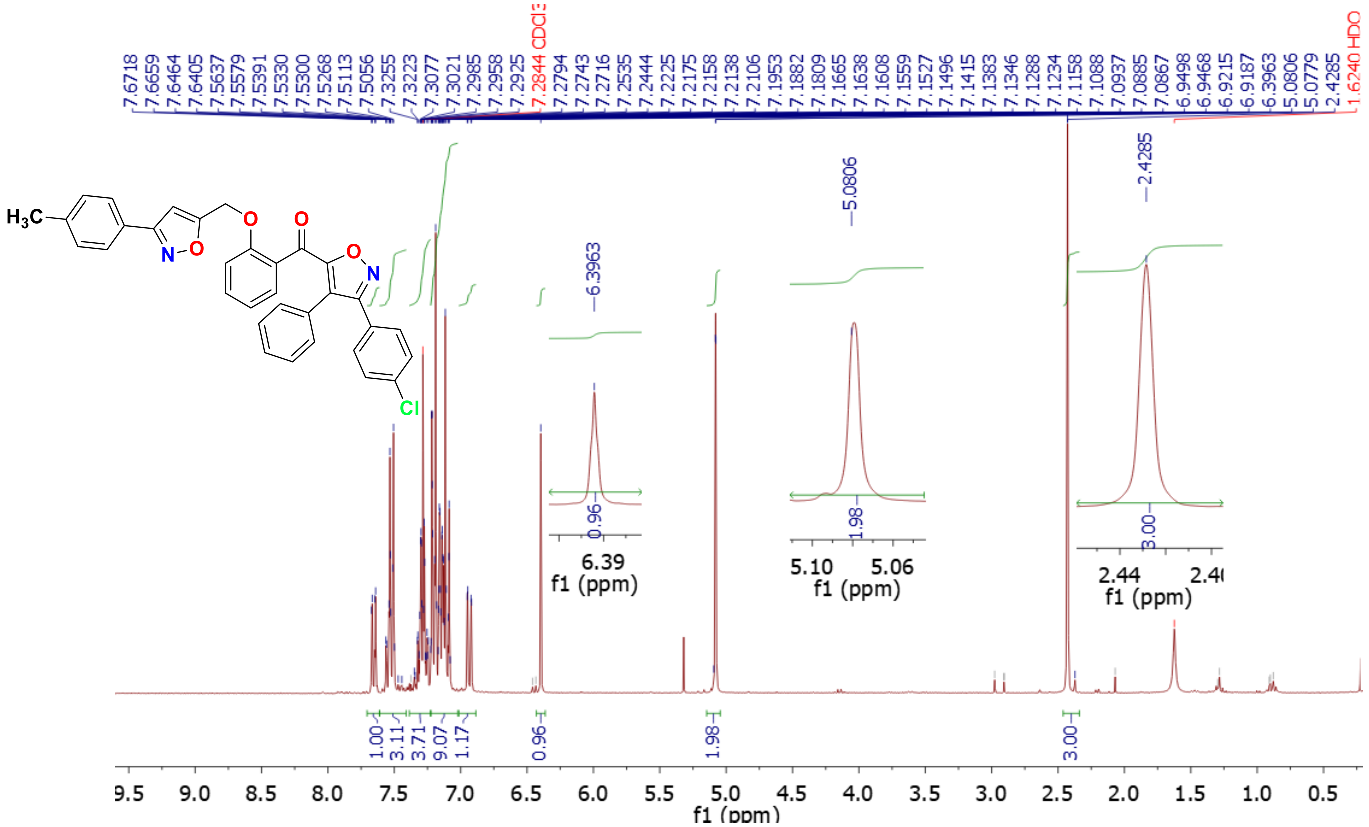

Figure S26. ^{1}H NMR spectrum (300 MHz, $CDCl_3$) of compound **5f**

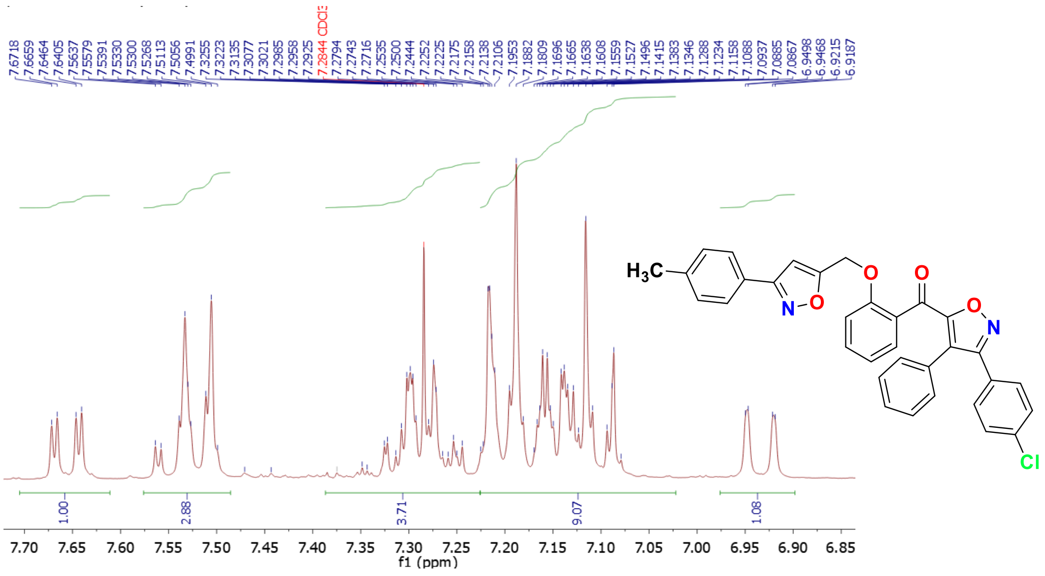

Figure S27. Aromatic enlarged region of ^{1}H NMR spectrum of compound **5f**

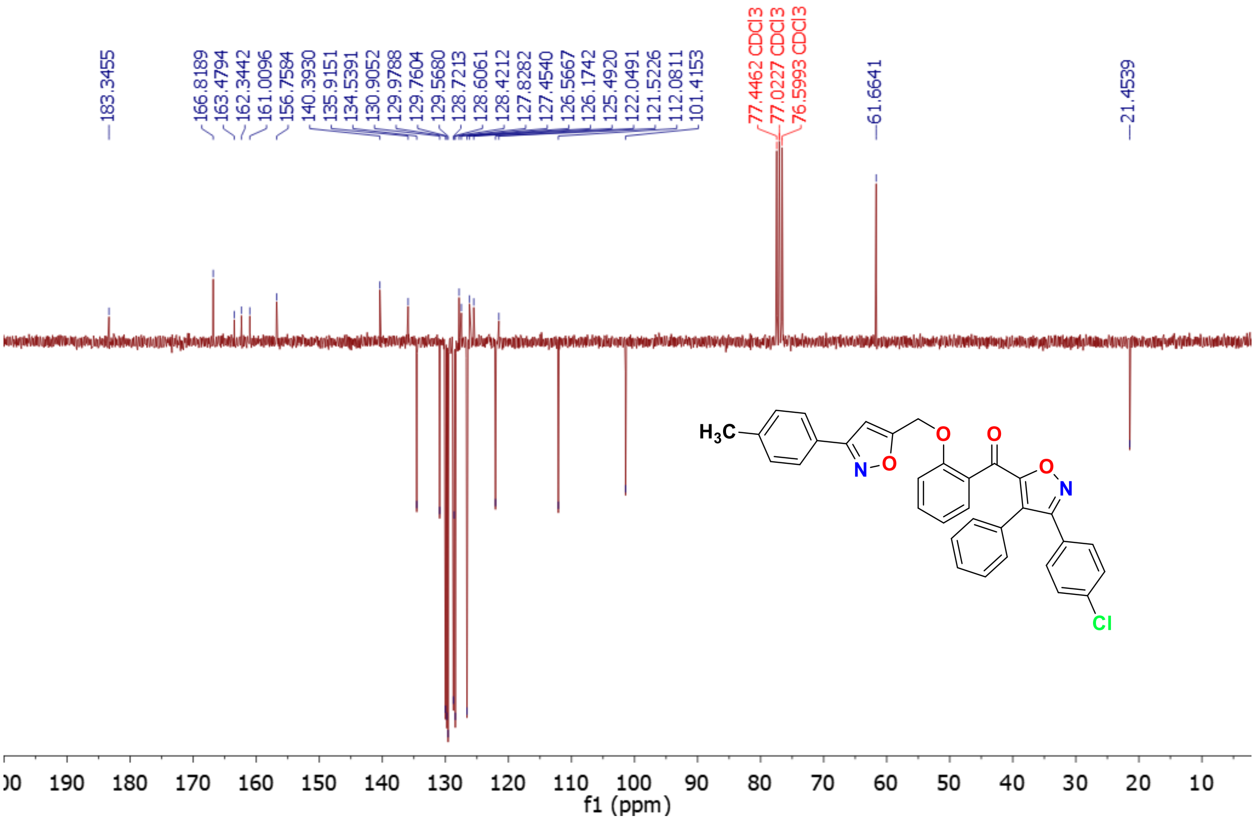

Figure S28. ^{13}C NMR spectrum (75 MHz, CDCl3) of compound **5f**

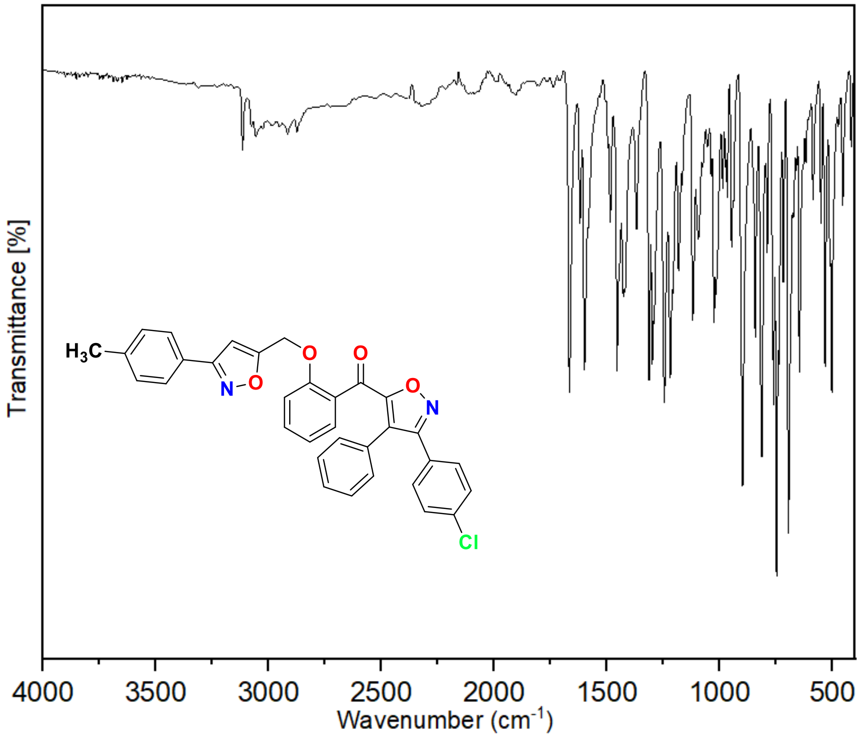

Figure S29. FT-IR spectrum of compound **5f**

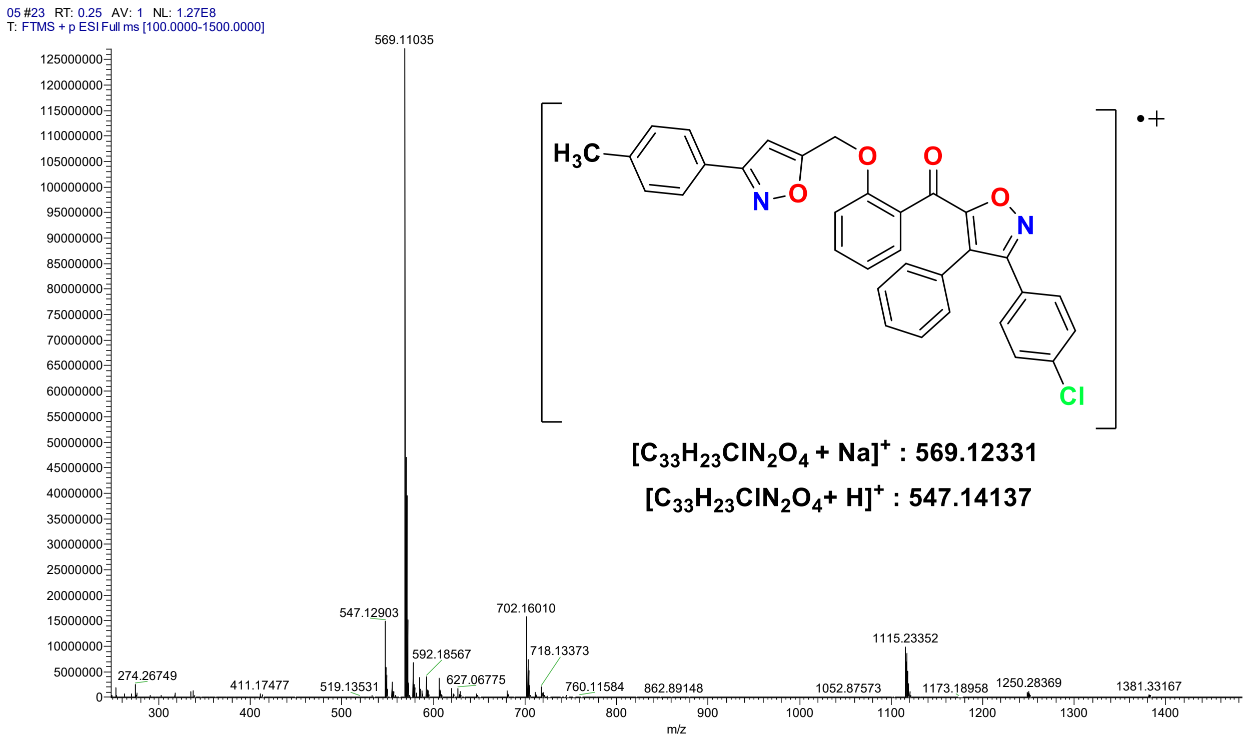

Figure S30. HRMS spectrum of compound **5f**

(3-(4-chlorophenyl)-4-phenylisoxazol-5-yl)(2-((3-(4-chlorophenyl)isoxazol-5-yl)methoxy)phenyl)methanone (5g):

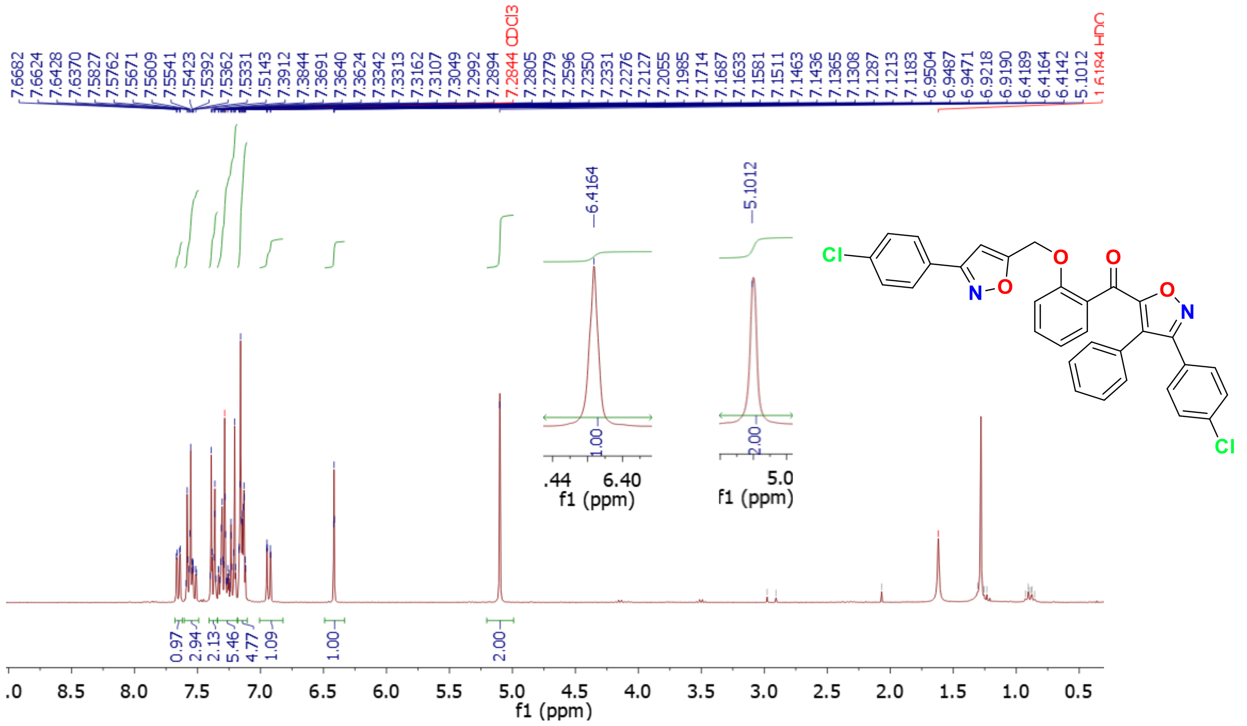

Figure S31. ^{1}H NMR spectrum (300 MHz, $CDCl_3$) of compound **5g**

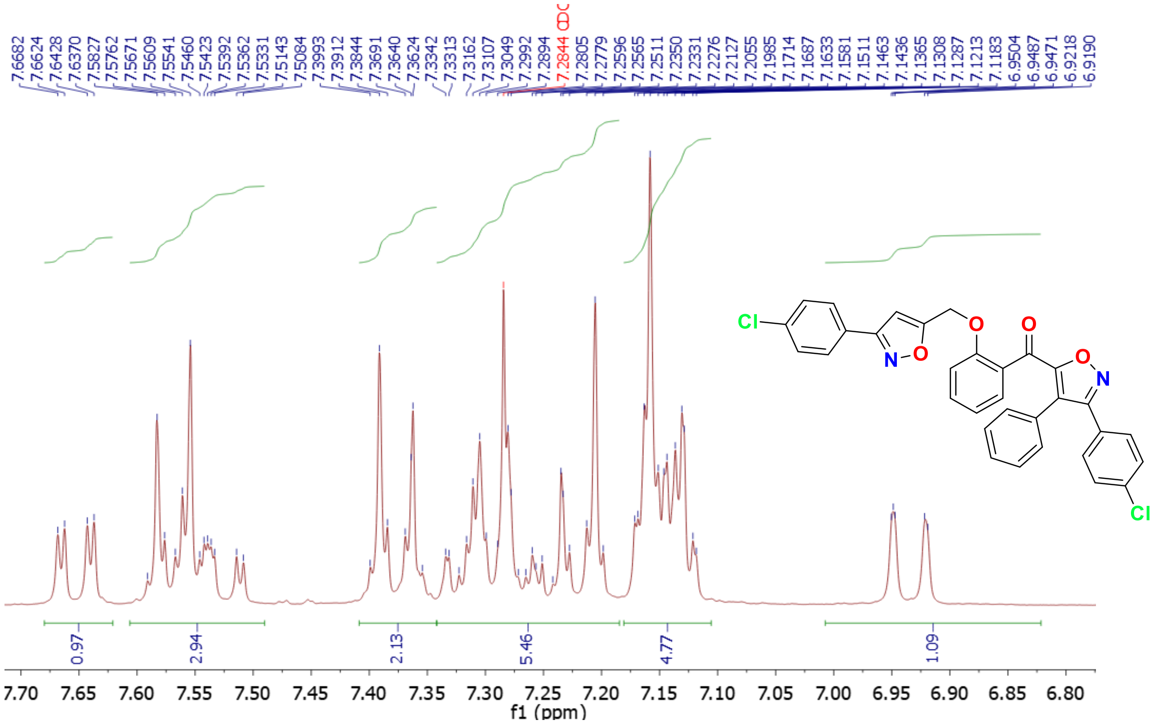

Figure S32. Aromatic enlarged region of ^{1}H NMR spectrum of compound **5g**

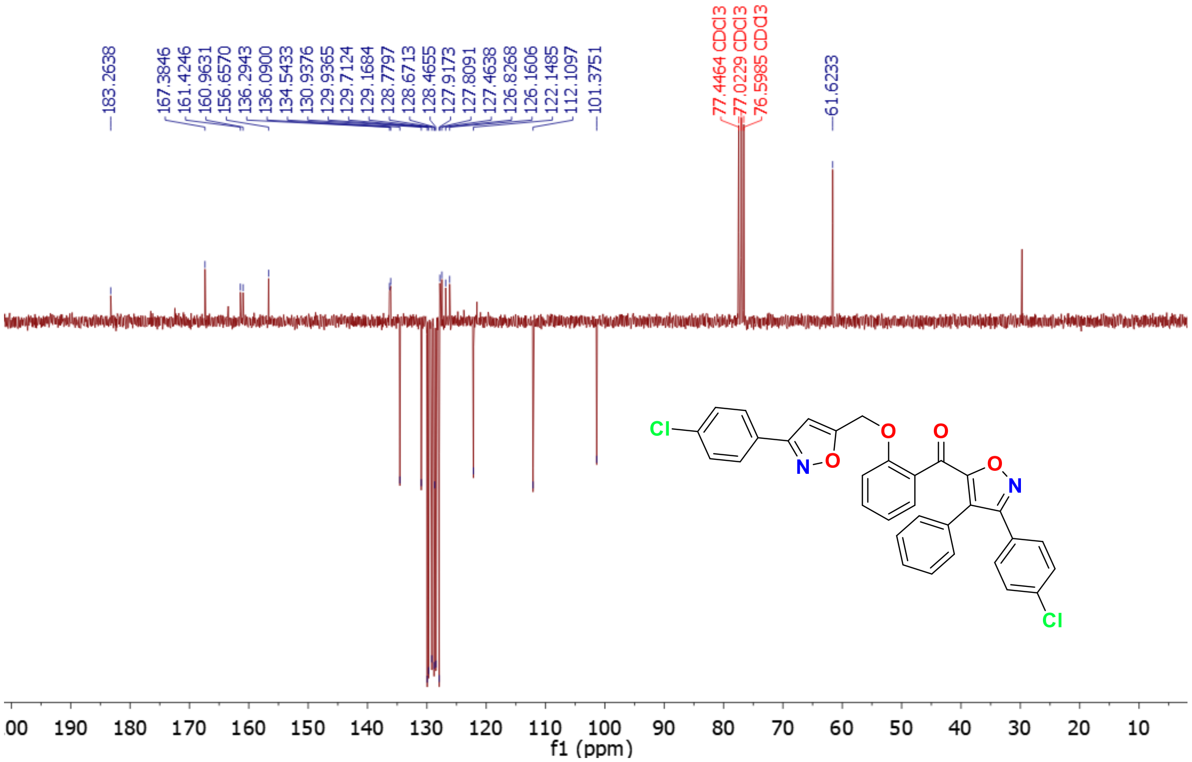

Figure S33. ^{13}C NMR spectrum (75 MHz, CDCl3) of compound **5g**

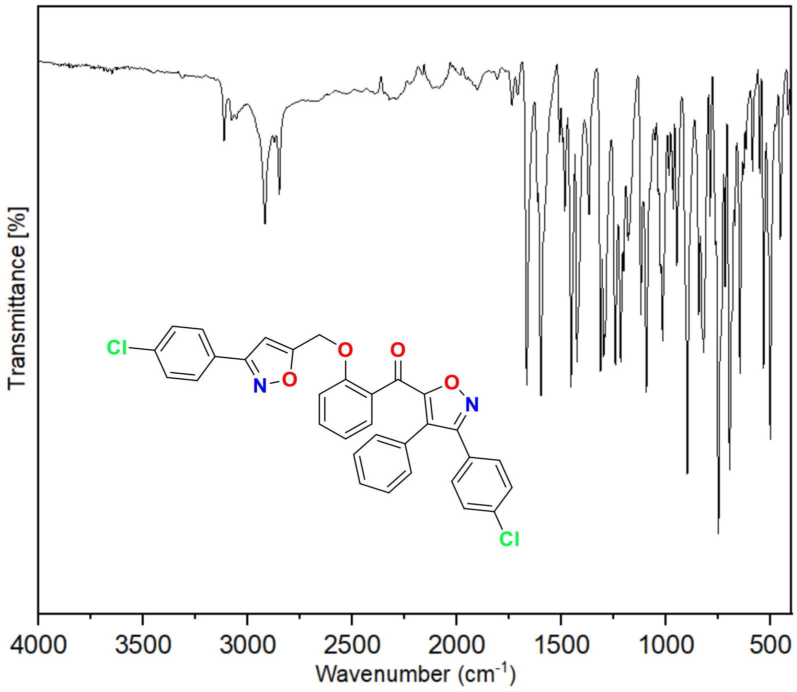

Figure S34. FT-IR spectrum of compound **5g**

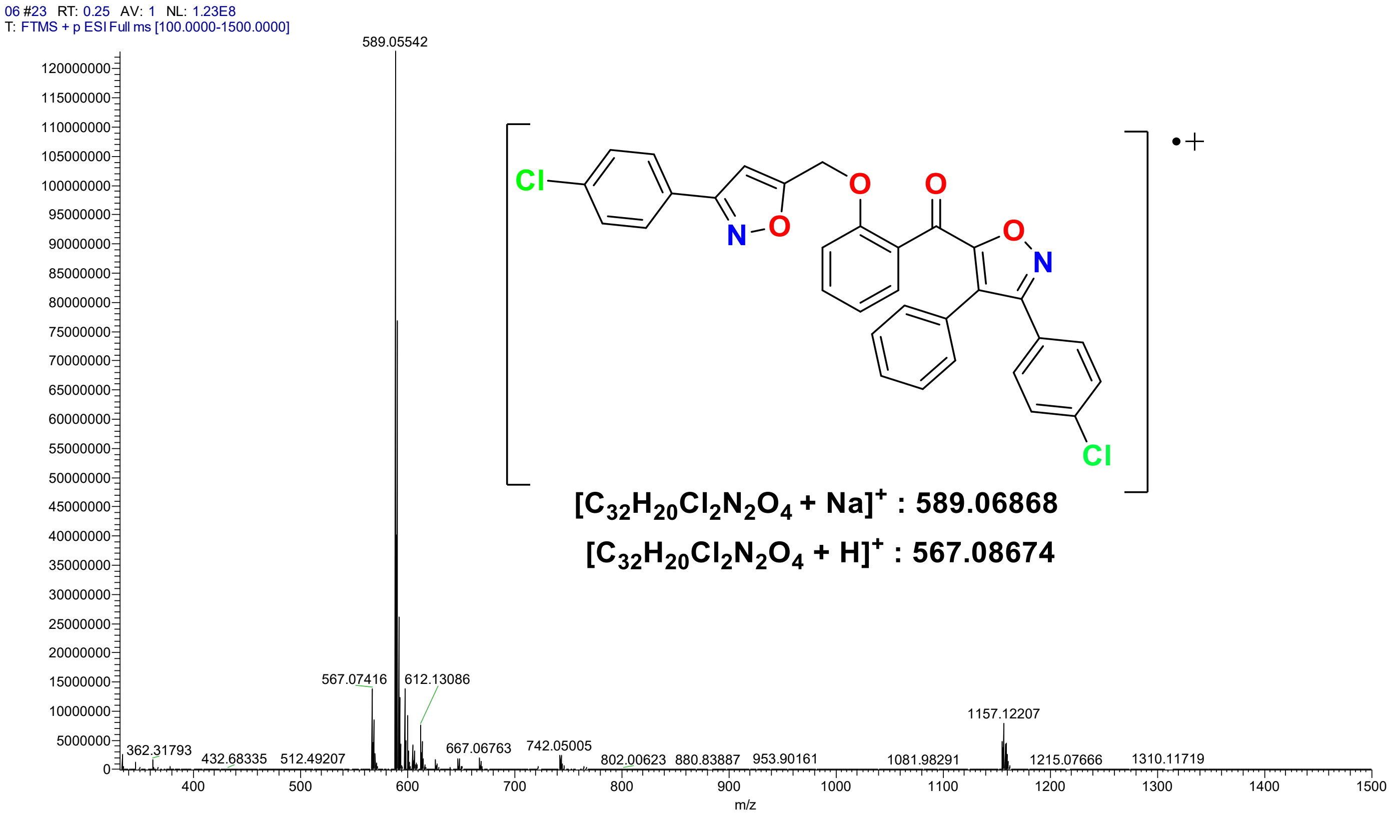

Figure S35. HRMS spectrum of compound **5g**

(3-(4-chlorophenyl)-4-phenylisoxazol-5-yl)(2-((3-(4-(trifluoromethyl)phenyl)isoxazol-5-yl)methoxy)phenyl)methanone (6h):

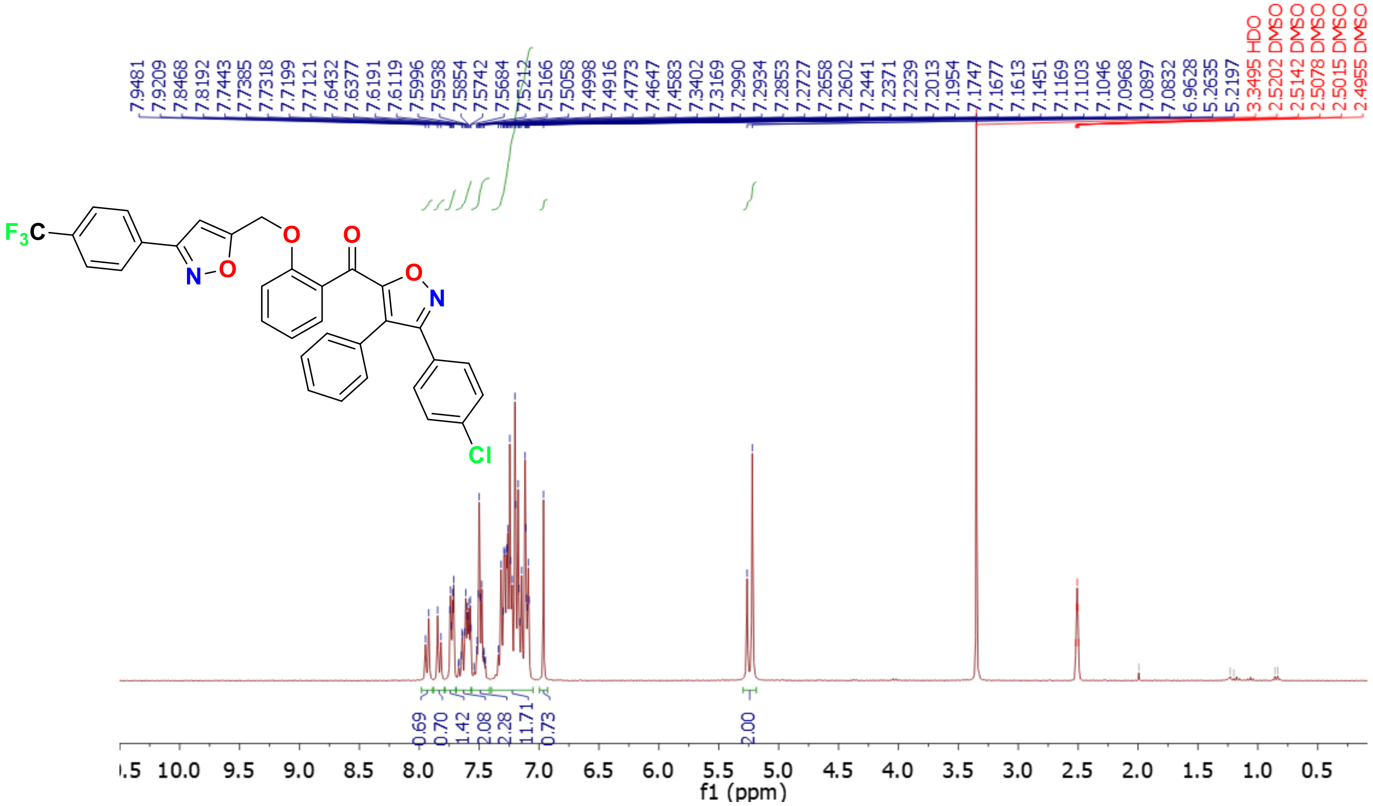

Figure S36. ^{1}H NMR spectrum (300 MHz, $CDCl_3$) of compound **5h**

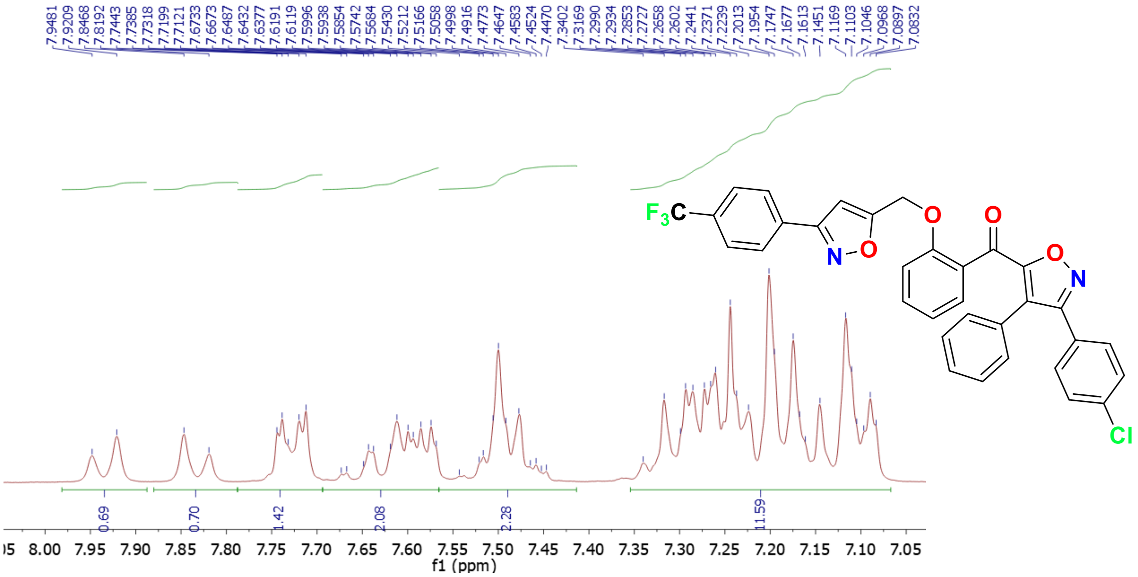

Figure S37. Aromatic enlarged region of ^{1}H NMR spectrum of compound **5h**

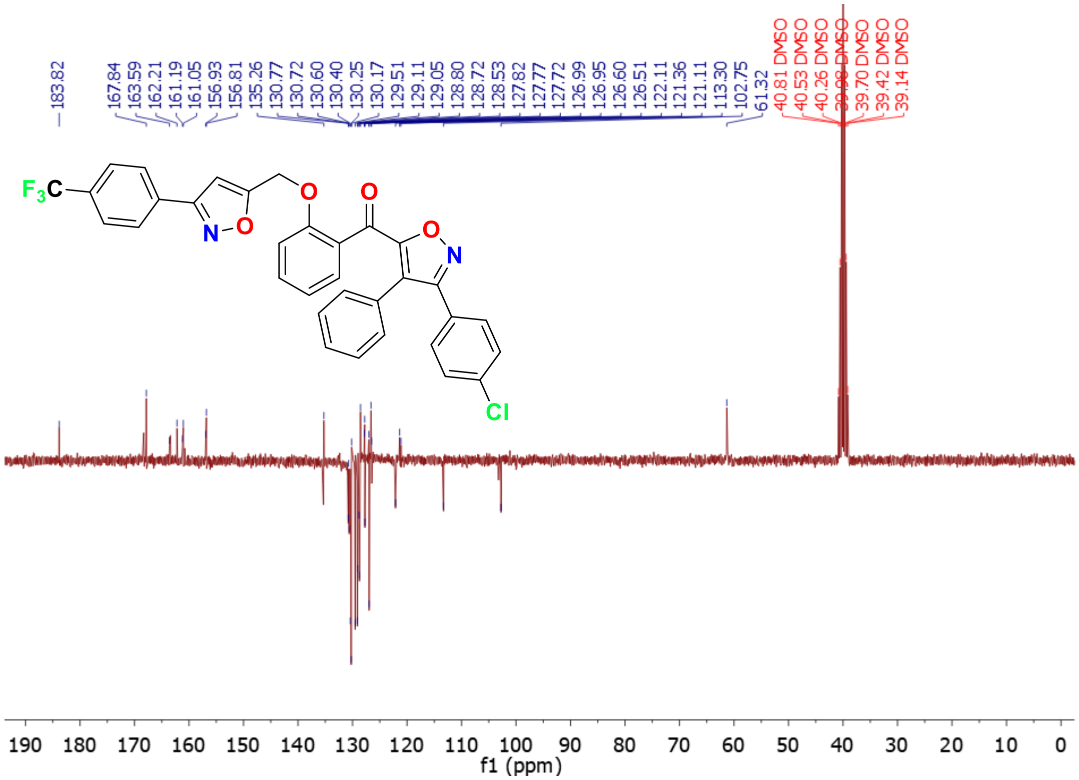

Figure S38. ^{13}C NMR spectrum (75 MHz, CDCl3) of compound **5h**

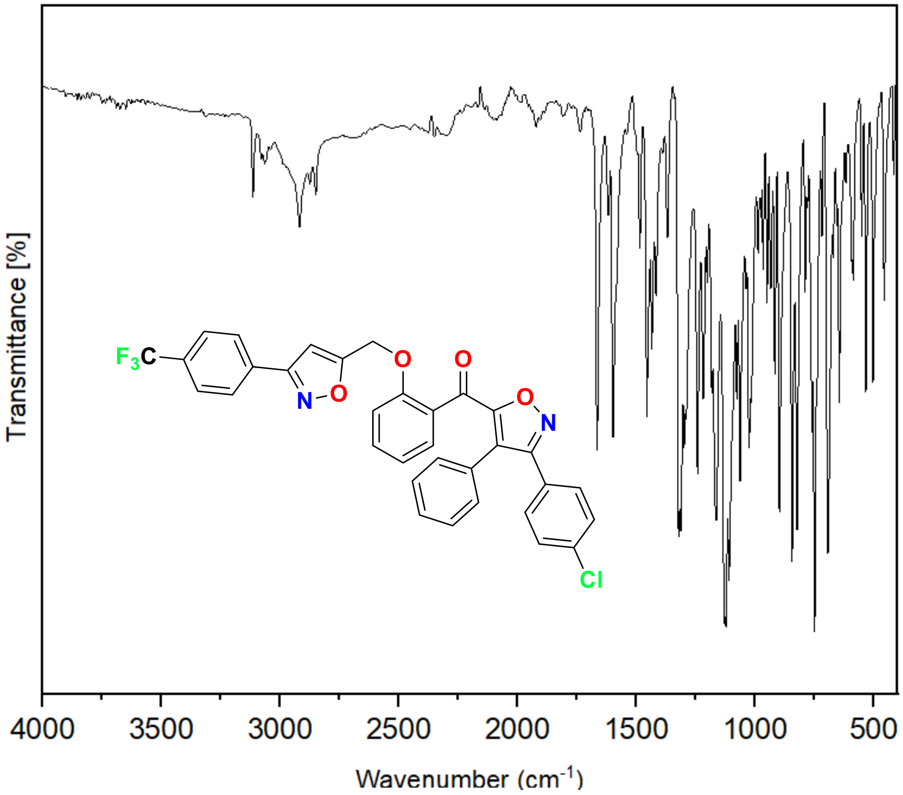

Figure S39. FT-IR spectrum of compound **5h**

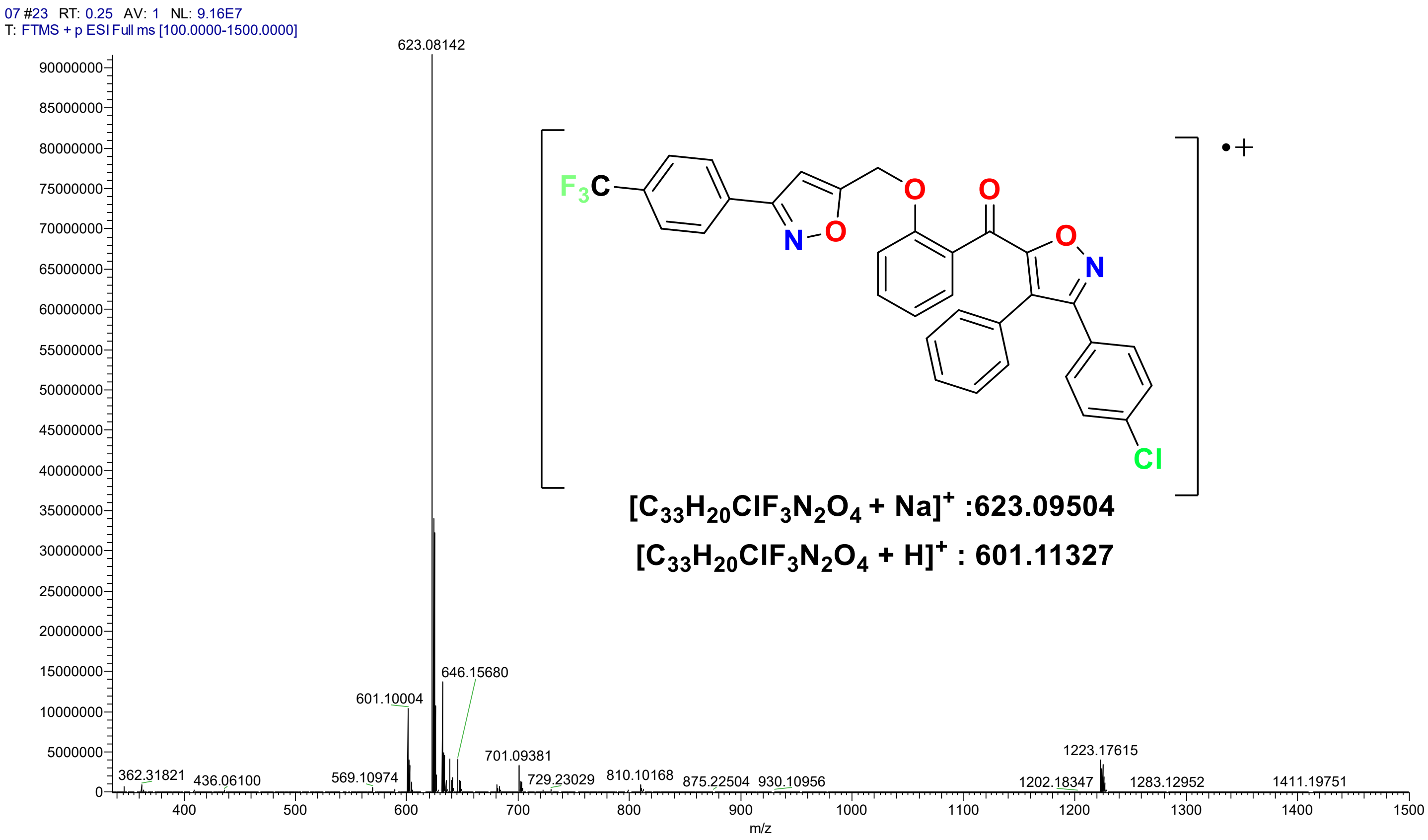

Figure S40. HRMS spectrum of compound **5h**

References:

1 C. Beney, A. Mariotte and A. Boumendjel, *Heterocycles*, 2001, **55**, 967–972.

2 S. Masoomi, E. Alipour, M. Ali, A. Reza and A. Shafiee, *Iran J Org Chem*, 2011, **3**, 733–736.

3 A. Arzine, O. Abchir, M. Chalkha, K. Chebbac, Y. Rhazi, N. Barghady, I. Yamari, A. E. L. Moussaoui, A. Nakkabi, M. Akhazzane, M. Bakhouch, S. Chtita and M. E. L. Yazidi, *Comput Biol Chem*, 2023, 107993.

4 A. Arzine, H. Hadni, K. Boujdi, K. Chebbac, N. Barghady, Y. Rhazi, M. Chalkha, A. Nakkabi, K. Chkirate, J. T. Mague, S. M. A. Kawsar, G. Al Houari, M. M. Alanazi and M. El Yazidi, *Molecules*, DOI:https://doi.org/ 10.3390/molecules29143366.

5 A. Arzine, S. A. Assou, F. Z. Guerguer, K. Boujdi, Y. Rhazi, N. Barghady, M. Chalkha, A. Nakkabi, K. Chkirate, J. T. Mague, M. Bakhouch, S. Chtita, H. Mabrouk, M. El Hassouni and M. El, *ChemistrySelect*, DOI:10.1002/slct.202405455.

6 M. A. Pfaller, V. Haturvedi, A. Espinel-Ingroff, M. A. Ghannoum, L. L. Gosey, F. C. Odds, J. H.

Rex, M. G. Rinaldi, D. J. Sheehan, T. J. Walsh and D. W. Warnock, *Reference Method for Broth Dilution Antifungal Susceptibility Testing of Yeasts ; Approved Standard — Second Edition Serving the World ' s Medical Science Community Through Voluntary Consensus*, 2002, vol. 22.

7 B. Veeraraghavan, Y. D. Bakthavatchalam and R. D. Sahni, *Infect Dis Ther*, 2021, **10**, 15–25.

8 S. Ait Assou, A. Grafov, D. Bousta, H. Bekkari and M. El Hassouni, *Sci African*, 2024, **25**, e02292.

9 G. Zengin, T. Arkan, A. Aktumsek, G. O. Guler and Y. S. Cakmak, *J Food Biochem*, 2013, **37**, 646–653.
